# The human ARF tumor suppressor senses blastema activity and suppresses epimorphic tissue regeneration

**DOI:** 10.7554/eLife.07702

**Published:** 2015-11-17

**Authors:** Robert G Hesse, Gayle K Kouklis, Nadav Ahituv, Jason H Pomerantz

**Affiliations:** 1 Department of Surgery, Division of Plastic Surgery, Program in Craniofacial Biology, University of California, San Francisco, San Francisco, United States; 2 Department of Bioengineering and Therapeutic Sciences and Institute for Human Genetics, University of California, San Francisco, San Francisco, United States; 3 Departments of Surgery and Orofacial Sciences, Division of Plastic Surgery, Program in Craniofacial Biology, Eli and Edythe Broad Center of Regeneration Medicine and Stem Cell Research, University of California, San Francisco, San Francisco, United States; California Institute of Technology, United States

**Keywords:** tumor suppressors, regeneration, ARF, evolution, Human, Zebrafish

## Abstract

The control of proliferation and differentiation by tumor suppressor genes suggests that evolution of divergent tumor suppressor repertoires could influence species’ regenerative capacity. To directly test that premise, we humanized the zebrafish p53 pathway by introducing regulatory and coding sequences of the human tumor suppressor *ARF* into the zebrafish genome. *ARF* was dormant during development, in uninjured adult fins, and during wound healing, but was highly expressed in the blastema during epimorphic fin regeneration after amputation. Regenerative, but not developmental signals resulted in binding of zebrafish E2f to the human *ARF* promoter and activated conserved ARF-dependent Tp53 functions. The context-dependent activation of ARF did not affect growth and development but inhibited regeneration, an unexpected distinct tumor suppressor response to regenerative versus developmental environments. The antagonistic pleiotropic characteristics of *ARF* as both tumor and regeneration suppressor imply that inducing epimorphic regeneration clinically would require modulation of ARF –p53 axis activation.

**DOI:** http://dx.doi.org/10.7554/eLife.07702.001

## eLife digest

Zebrafish are capable of remarkable feats of regeneration in which damaged or lost body parts can be replaced with exact replicas of the original. Humans cannot do this; so one major goal of regenerative medicine is to understand the basis of these differences. This could make it possible to regenerate damaged limbs, heart muscle and other tissues in people.

A group of genes called tumor suppressor genes are critical for protecting us from cancer. However, they also limit the ability of cells to grow and divide, which are both important for regeneration processes. Differences in how organisms use tumor suppressor genes could possibly contribute to their differing abilities to regenerate body parts.

A tumor suppressor gene called *ARF* plays an important role in protecting mammals from cancer, but it is not found in zebrafish and other animals that can regenerate body parts. Hesse et al. introduced the human *ARF* gene into zebrafish to investigate whether it has any effect on tissue regeneration. The experiments show that *ARF* is silent in growing and uninjured fish, but it is activated when it detects regeneration occurring after an injury. *ARF* drastically inhibits this process to the point that fish with the *ARF* gene cannot regenerate damaged tissue. The findings suggest that *ARF* mistakenly identifies regeneration as the formation of a tumor; therefore therapies that aim to induce regeneration in people may need to control the activity of this gene.

Hesse et al.’s findings show that the specific tumor suppressor genes an organism has can affect its ability to regenerate. Future challenges will be to understand the situations in which *ARF* is able to interfere with tissue regeneration in mammals, and to learn how to manipulate this process.

**DOI:** http://dx.doi.org/10.7554/eLife.07702.002

## Introduction

Urodele amphibians and teleost fish are unique among vertebrates in that they possess the ability to regenerate injured complex structures such as limbs, fins, jaws, and heart by epimorphic regeneration (Morgan, 1901; Brockes and Kumar, 2008; Poss, 2010). For example, zebrafish fin regeneration proceeds through steps that include wound healing, blastema formation, and regenerative outgrowth to faithfully restore preinjury structures and size of the fin (Poss et al., 2003). In such highly regenerative species, the blastema consists of a heterogeneous pool of highly proliferative mesenchymal cells that gives rise to the large amount of new tissue in the regenerate (Knopf et al., 2011; Tu and Johnson, 2011). In contrast, absence of a proliferative blastema is a prominent feature of most mammalian solid tissue injury responses (Muneoka et al., 2008; Straube and Tanaka, 2006). An open question in biology is how cellular mechanisms controlling proliferation affect the blastema and whether they have evolved to contribute to divergent regenerative capacities among vertebrate species.

Tumor suppressor genes control the proliferative and differentiated state of cells, and many are also developmental regulators critical for normal formation of tissues (Jacks et al., 1992; Berman et al., 2008). The complex and precisely controlled proliferation and differentiation that occurs during epimorphic regeneration likely requires similar machinery, and as a group, tumor suppressors are probably necessary for well-orchestrated regeneration to occur (Pomerantz and Blau, 2013). For example, in eukaryotes the retinoblastoma gene *Rb1* regulates the G1/S transition by sequestering E2f transcription factors, and it controls cellular differentiation by associating with chromatin modifiers to regulate activity of tissue-specific transcription factors. Therefore, the role of *Rb1 *in tumor suppression is likely less important from an evolutionary standpoint than its ancient broad functions in regulating cellular differentiation and tissue formation. In contrast, the mammalian gene *Cdkn2a* is an essential tumor suppressor in mice and humans, but it is dispensable for mammalian development and tissue formation (Serrano et al., 1996). In mammals, *Cdkn2a *encodes two structurally unrelated proteins translated via alternate reading frames, p16Ink4a and Arf, each of which is a tumor suppressor (Chin et al., 1998; Sherr, 2006). While p16Ink4a is a cyclin-dependent kinase inhibitor (CKI) that functions upstream of Rb1, Arf exerts its tumor suppressor function by responding to inappropriate Rb pathway signaling above a presumed threshold (Lowe and Sherr, 2003). When induced, it stabilizes and activates Tp53 by binding and sequestering Mdm2, an E3 ubiquitin ligase and negative regulator of Tp53 (Pomerantz et al., 1998; Weber et al., 1999). Depending on the context, stabilized Tp53 either promotes cell cycle arrest or apoptosis. In addition to canonical Tp53-dependent functions, Arf has other important functions including controlling ribosome biogenesis and responding to oxidative stress (Sherr, 2006; Weber et al., 2000; Damalas et al., 2011; Menendez et al., 2003). The resulting general function of Arf is to maintain the postmitotic state, and we have previously shown that suppression of Arf in the context of compromise of the Rb pathway results in dedifferentiation and proliferation of mammalian muscle cells in culture (Pajcini et al., 2010). Unlike in development or in regeneration of epithelial and hematopoietic tissues, reversal of the postmitotic state and dedifferentiation also occur in lower vertebrate epimorphic regeneration scenarios that involve a blastema. Regulation of Tp53 has recently been shown to be important during epimorphic regeneration, where it is downregulated during blastema formation (Yun et al., 2013). Although cell cycle reentry of postmitotic cells and dedifferentiation are characteristics of malignant transformation which tumor suppressor mechanisms oppose, why these two processes are permitted to occur in the context of intact tumor suppressor mechanisms during epimorphic regeneration is unknown.

How evolution of the central cellular growth and tumor suppressor pathways impacts regenerative capacity is poorly understood. The advent of somatic stem cells in metazoans is thought to have enabled the formation of new types of cancer, thus requiring advanced tumor suppressor mechanisms (Belyi et al., 2010; Pearson and Sanchez Alvarado, 2008). Among metazoan species, including vertebrates, selective pressures such as different physiologies and environmental exposures undoubtedly continue to apply pressure to generate species-specific tumor suppressor repertoires. For some tumor suppressor genes such as *Tp53*, multiple family members have evolved to carry out certain differentiation functions separately from tumor suppression. For others, such as *Arf*, a single member has evolved and exists in a limited number of species. Whether such differences in turn relate to distinct regenerative capacities remains unknown.

**Figure 1.**
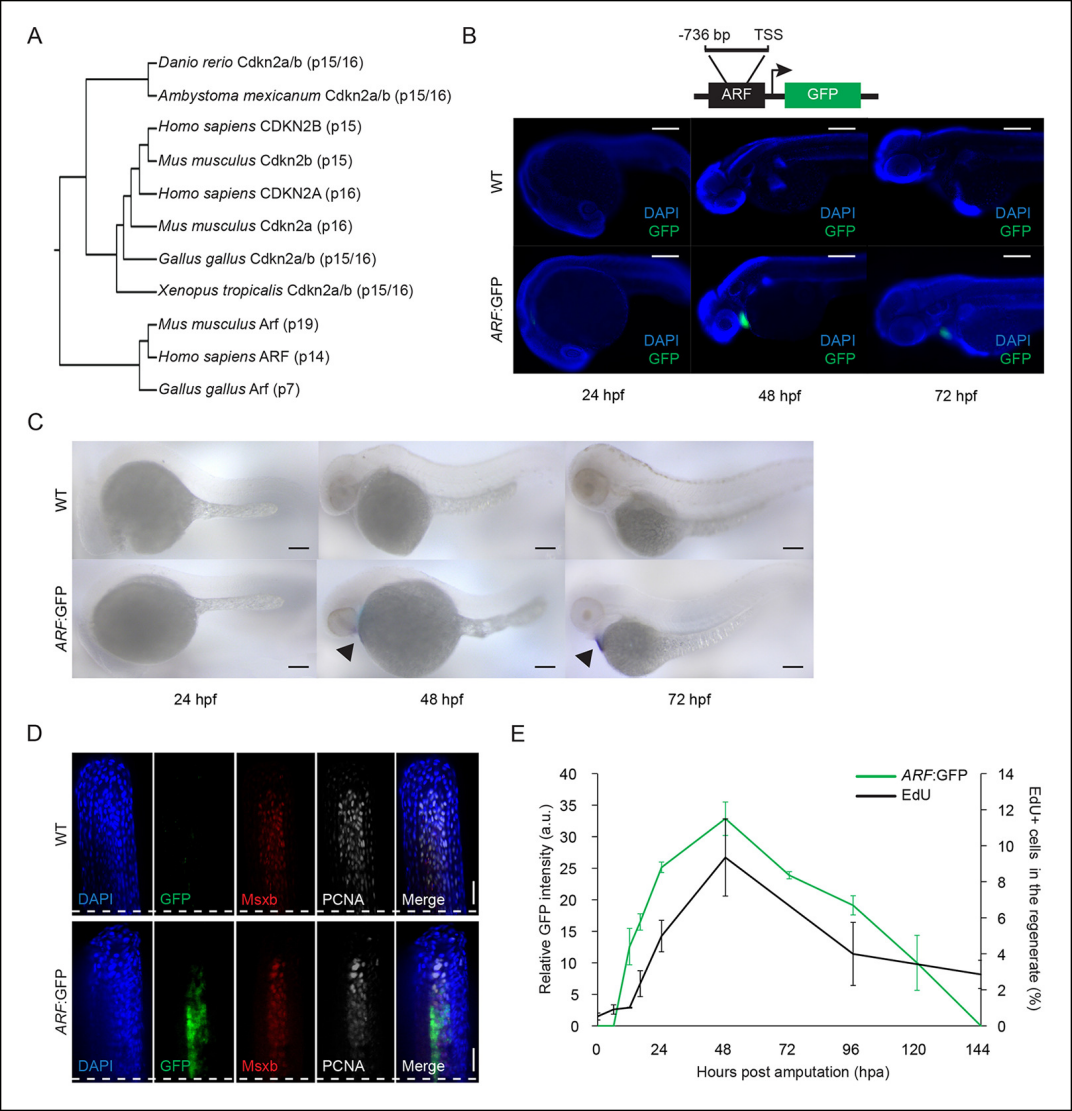
*ARF*, not normally present in highly regenerative vertebrates, is specifically activated in blastemas of *ARF* transgenic zebrafish. (**A**) Comparison of amino acid sequences of proteins produced by the Cdkn2a/b loci of zebrafish (*Danio rerio*), amphibians including the axolotl (*Ambystoma mexicanum*) and western clawed frog (*Xenopus tropicalis*), chickens (*Gallus gallus*), and mammals including the mouse (*Mus musculus*) and human (*Homo sapiens*). While Cdkn2a and Cdkn2b are conserved and encode Ink4 orthologs, Arf evolved recently and orthologs do not exist in highly regenerative vertebrates including teleost fish and urodele amphibians. (**B**) Schematic of transgene expressing cytoplasmic Green fluorescent protein (GFP) under the control of the human ARF promoter (top). The promoter consists of human regulatory sequences 736 bp upstream of the transcriptional start site (TSS) of *ARF*. Immunostaining (wide-field images) for GFP at 24 hpf, 48 hpf, and 72 hpf in wild type (WT) and ARF:GFP embryos (bottom). Scale bars: 200 μm. GFP expression is visible in the hearts of transgenic fish due to presence of a separate transgene used for selection (*cmlc2*:GFP). (**C**) Whole-mount in situ hybridization for GFP at 24 hpf, 48 hpf, and 72 hpf in WT and *ARF*:GFP embryos. Scale bars: 100 μm. Alkaline phosphatase staining is detected in the hearts of transgenic fish (arrow heads) because of the selection transgene as in (**B**). (**D**) Confocal images of coronal vibratome sections immunostained for GFP, Msxb, and Proliferating cell nuclear antigen (PCNA) at 2 dpa in WT and *ARF*:GFP fins. Scale bars: 50 μm. GFP expression is induced in the proliferative blastema of the regenerate, but it is not expressed in the surrounding epithelium. White dashed lines represent amputation planes. (**E**) GFP intensity (green line) in the regenerates of *ARF*:GFP transgenic fish relative to WT fish after amputation. The black line represents the percentage of EdU + cells in the regenerates of WT fish after amputation (N=3; secondary axis). Figure supplement 1 shows in vitro *ARF* promoter assays. Figure supplement 2 shows additional images for panels **B, D**, and **E**. Figure supplement 3 shows wound healing in WT and *ARF*:GFP fins. Results are shown as mean ± standard deviation. hpa: Hours post amputation. **DOI:**http://dx.doi.org/10.7554/eLife.07702.003

Although tumor suppressors are generally highly conserved in eukaryotes, *Arf *is unusual in that it is poorly conserved in non-mammalian lineages (Figure 1A). The *Cdkn2a/b* locus of teleost fish, including zebrafish (*Danio rerio*) and fugu (*Takifugu rubripes*) (Gilley and Fried, 2001), exists as a single protein-producing unit that only encodes for a CKI. During evolution, *Cdkn2a* and *Cdkn2b* developed into two separate but related genes encoding for biochemically related CKIs. Arf is not a CKI and is not closely related to either Cdkn2a or Cdkn2b. Arf is thought to be the product of a genetic duplication caused by either an insertion or transposition into the *Cdkn2a/b* locus (Gil and Peters, 2006). Of the highly regenerative species for which genomes have been completely sequenced, none possess an ortholog of *Arf *(Figure 1A) (Flicek et al., 2014; Karolchik et al., 2014). The earliest documented ortholog of *Arf* exists in the chicken genome (Kim et al., 2003). This restricted representation coupled with *ARF* functions of responding to a high threshold of proliferative signaling and inhibiting dedifferentiation (Pajcini et al., 2010; Sherr, 2006) is compatible with a hypothesis that the presence of *Arf *could impact regenerative capacity.

**Figure 1—figure supplement 1.**
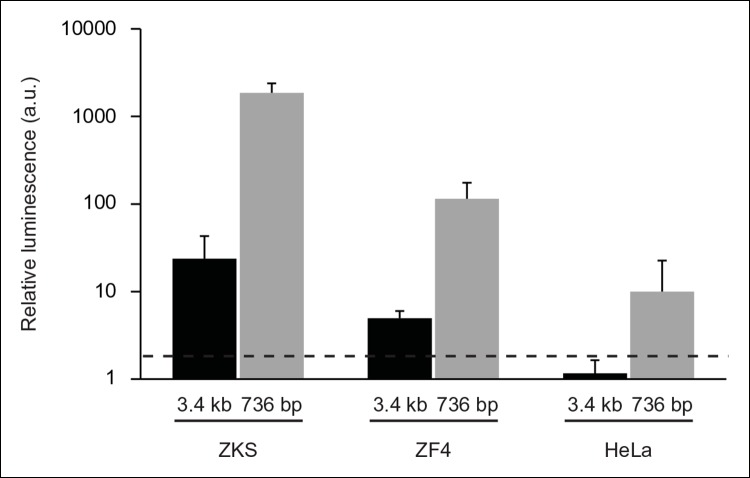
In vitro analysis of *ARF *promoter constructs in zebrafish and human cells. Representative luciferase reporter data of three replicates: relative luminescence generated by *ARF* promoter-firefly luciferase reporter constructs transfected into zebrafish (ZKS, ZF4) and human (HeLa) cells. Two *ARF* promoter-reporter constructs were tested; one contained sequences up to 3.4 kb upstream of the TSS of *ARF* (3.4 kb), while the other contained sequences up to 736 bp upstream of the TSS of *ARF* (736 bp). Relative luminescence was measured by normalizing firefly luciferase values to those detected from a Renilla luciferase construct used as a transfection efficiency control. The relative luminescence values were then normalized to those of the negative control construct, pcDNA. Any values above 2 (black dashed line) are significant (N = 3; pcDNA = 1; p<0.05). Results are shown as mean ± standard deviation. ZKS: Zebrafish kidney stromal. **DOI:**http://dx.doi.org/10.7554/eLife.07702.004

**Figure 1—figure supplement 2.**
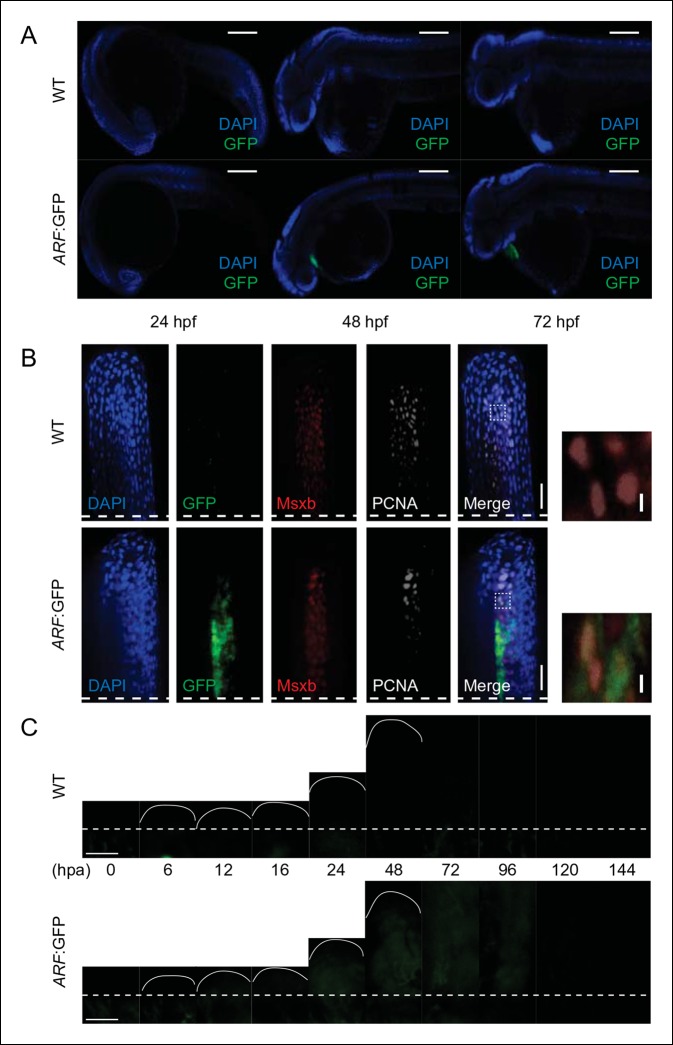
GFP reporter activity in WT or *ARF*:GFP zebrafish at multiple developmental and regenerative time points. (**A**) Immunostaining (sagittal confocal images) for GFP at 24 hpf, 48 hpf, and 72 hpf in WT and *ARF*:GFP embryos. Scale bars: 200 μm. GFP expression is restricted to the hearts of transgenic fish due to presence of a separate transgene used for selection (*cmlc2*:GFP). (**B**) Confocal images from Figure 1 of coronal vibratome sections immunostained for GFP, Msxb, and PCNA at 2 dpa in WT and *ARF*:GFP fins. Scale bars: 50 μm. Included to the right of the figure are insets showing Msxb +, PCNA +, GFP- blastema cells in WT fins and cytoplasmic GFP expression in Msxb +, PCNA + blastema cells in *ARF*:GFP fins (white dashed boxes). DAPI is excluded from the inset images to improve clarity of costaining. Scale bars: 10 μm. (**C**) Wide-field epifluorescent images of WT and *ARF*:GFP fins at multiple time points during fin regeneration. GFP intensity of individual *ARF*:GFP images was evaluated relative to that of WT images at the same time points, and the resulting values were plotted in Figure 1E. There is a small amount of detectable autofluorescence below the amputation plane in the regenerating wild-type and transgenic fins. Scale bars: 100 μm. Dashed lines represent amputation planes. GFP: Green fluorescent protein; hpa: Hours postamputation; PCNA: Proliferating cell nuclear antigen; WT: Wild type. **DOI:**http://dx.doi.org/10.7554/eLife.07702.005

**Figure 1—figure supplement 3.**
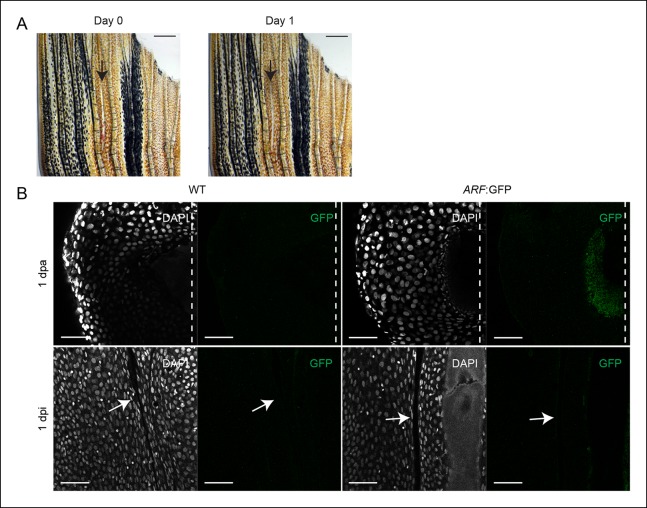
*ARF* is not activated during wound healing in the absence of a blastema. (**A**) At day 0, dorsal fin lobes were wounded (interray laceration, 0 dpi), while ventral fin lobes were amputated (0 dpa). At day 1, GFP expression was assayed in the healing (dorsal) and regenerating (ventral) fins. Scale bars: 0.5 mm. (**B**) Representative images (sagittal confocal images) of cytoplasmic GFP expression in WT and *ARF*:GFP fins that were either amputated (1 dpa) or wounded (1 dpi). GFP is only detected in *ARF*:GFP fins that have been amputated (N = 5). Scale bars: 50 μm. Arrows point to the interray wound. Dashed lines represent amputation planes.GFP: Green fluorescent protein; WT: Wild type. **DOI:**http://dx.doi.org/10.7554/eLife.07702.006

In this study, we used transgenesis to examine the activity of human *ARF* in the context of zebrafish fin regeneration. We showed that *ARF* activated zebrafish Tp53 functions to restrict cellular proliferation and induced apoptosis, which caused a marked suppression of fin regeneration after injury. Remarkably, the human *ARF* regulatory sequences are dormant throughout zebrafish development but induce ARF expression specifically during regeneration after injury. These findings provide experimental evidence that species-specific tumor suppressors can impact tissue regeneration potential.

## Results

### Survey of *ARF* orthologs in genomes of highly and poorly regenerative vertebrates 

Using the Ensembl Genome Browser (Flicek et al., 2014), the University of California, Santa Cruz (UCSC) Genome Browser (Karolchik et al., 2014), and Sal-Site (Smith et al., 2005), we analyzed the Ink4b-Arf-Ink4a locus in the genomes of six different vertebrate species including highly regenerative (teleost fish and urodele amphibians) and poorly regenerative (avians and mammals) vertebrates (Monaghan and Maden, 2013). Our analysis confirms prior reports (Kim et al., 2003) that an ARF ancestor exists in chickens. We found that in contrast to Ink4 orthologs, which are pervasive throughout vertebrate genomes, ARF orthologs are not present in the genomes of highly regenerative vertebrates (Figure 1A). The results of this analysis, while not directly demonstrating an association of ARF with regeneration, support the hypothesis and prompted our investigation.

### Context-specific activation of* ARF* by regenerative signals in the zebrafish blastema

To investigate how the *ARF *gene responds to environmental cues, we generated reporter fish in which green fluorescent protein (GFP) is expressed under the control of the human *ARF *promoter (Tg (*ARF*:GFP) or *ARF*:GFP) (Figure 1B, top). In mammals, ARF expression is regulated by a promoter that contains several putative E2F binding sites, and *ARF* expression can be regulated by free E2F levels above a threshold (Gil and Peters, 2006). The ARF promoter has previously been empirically defined (del Arroyo et al., 2007) and no other regulatory sequences or enhancers have been described to date. We first confirmed that the human *ARF* promoter can function in zebrafish cells *in vitro* in transfection experiments using previously described firefly luciferase reporter constructs (del Arroyo et al., 2007). Experiments were performed with ZF4 and zebrafish kidney stromal (ZKS) (Stachura et al., 2009) cells with HeLa cells used as a positive control since they express high levels of endogenous ARF (Figure 1—figure supplement 1). These assays confirmed that the 736 base pair (bp) promoter is active in HeLa cells and in zebrafish lines. Therefore, we chose the 736 bp genomic fragment encompassing the human *ARF* promoter to generate *ARF *transgenics that mimic regulation of the human *ARF* gene. Tol2-mediated transgenesis (Kwan et al., 2007) was used to generate *ARF*:GFP fish, and transgenic fish were detected using cardiac GFP expression driven by a separate *cmlc2*:GFP cassette on the transgene.

We monitored expression of GFP driven by the human *ARF* promoter during normal development and in adult fish after injury and during regeneration. To determine if* ARF*:GFP is active during organogenesis in the zebrafish embryo, we assayed GFP expression at three developmental time points, 24, 48, and 72 hr postfertilization (hpf). We were unable to detect *ARF*:GFP expression in the embryo head, body, or tail by wide field epi or confocal immunofluorescence indicating that the *ARF *promoter is silent at these developmental stages (Figure 1B, Figure 1—figure supplement 2A). GFP expression in the heart driven by the *cmlc2* promoter serves as an internal control. To confirm that our analysis was not significantly compromised by limits of detection, we also performed *in situ* hybridization for GFP transcripts (Figure 1C). The *in situ* hybridization results confirmed the immunofluorescence analysis and indicate that *ARF *is silent or minimally expressed during development, including the developing tail fin region.

To investigate *ARF* activation during regeneration, *ARF*:GFP regenerates were assessed at the time of amputation and then at time points during which wound healing, blastema formation, and outgrowth of regenerating fins occurs (Poss et al., 2003). *ARF*:GFP transgenic fish regenerated their fins normally. In stark contrast to development, *ARF*:GFP was induced and highly expressed in the blastema of regenerating adult fins. GFP was specifically detected in *ARF*:GFP fins after amputation, and GFP colocalized with Msxb and proliferating cell nuclear antigen (PCNA) expressing cells (Figure 1D, Figure 1—figure supplement 2B). GFP was not detected in the surrounding epithelium. This observation indicates that the *ARF* promoter is active in at least a subset of Msxb + blastema cells. GFP signal in the regenerate was first detected at 12 hr postamputation (hpa), peaked at 48 hpa, and then declined to undetectable levels within 6 days (Figure 1E, Figure 1—figure supplement 2C). To correlate GFP expression with proliferation in *ARF*:GFP fins, we assessed EdU incorporation in regenerates at the above time points. There is a low level of proliferation in uninjured fins (0 hpa), but proliferation quickly increases to maximal levels within 48 hpa and then decreases (Figure 1E). GFP expression mirrored proliferative changes, suggesting that *ARF* detects and responds to high proliferative signaling in the regenerate.

To further examine the specificity of *ARF* regulation, we examined the response to the creation of an epithelial laceration wound. In this interray wound model, healing occurs without regeneration or blastema formation (Gauron et al., 2013). An epithelial wound was created in the dorsal fin lobe (Figure 1—figure supplement 3A) and the ventral fin lobe of the same fish was amputated. GFP expression was evaluated 1 day postinjury (dpi) (Figure 1—figure supplement 3B). As expected, GFP was detected in the forming ventral blastema. However, GFP was undetectable in the healing wound. These distinct *ARF *responses to development and to the two different forms of injury indicate that *ARF* specifically senses and responds to signals particular to the regeneration environment that differ significantly from those present during wound healing or in the highly proliferative environment of developmental organogenesis.

### Zebrafish E2f1 binds the human ARF promoter specifically in the context of Rb hyperphosphorylation during regeneration

**Figure 2.**
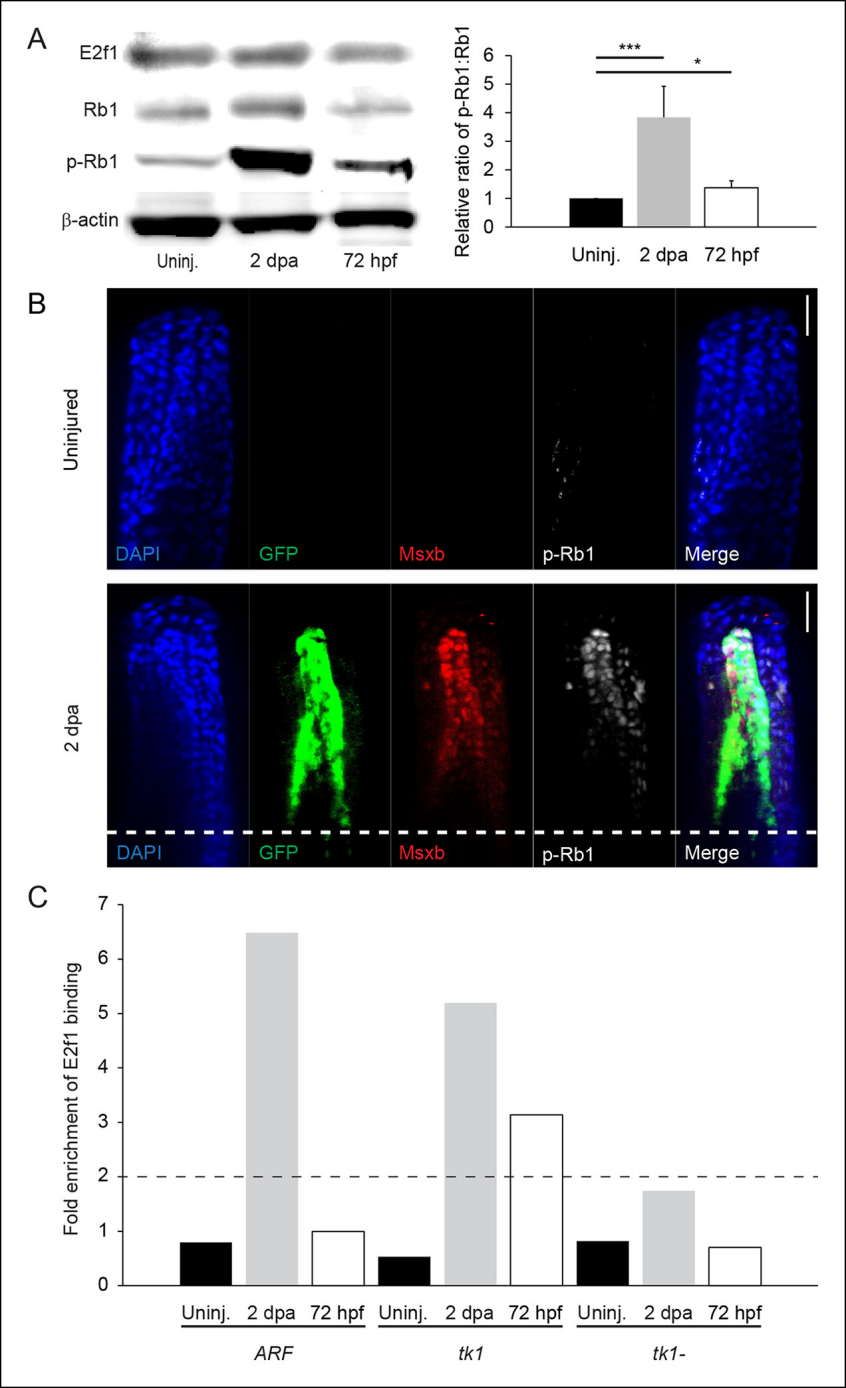
Rb1 hyperphosphorylation and E2f1 binding of the human ARF promoter in the blastema during regeneration. (**A**) Representative Western blot of three experimental replicates of Rb pathway components, E2f1, Rb1, and hyperphosphorylated Rb1 (p-Rb1), before injury (uninj.), at 2 dpa, and during embryogenesis at 72 hpf (left). Quantification of p-Rb1 and Rb1 levels normalized to β-Actin and relative to uninjured tissue. Results are from three independent biological replicate experiments and are shown as mean ratios ± standard deviation. *p<0.05; ***p<0.001 (right). (**B**) Confocal images of coronal vibratome sections immunostained for Green fluorescent protein (GFP), Msxb, and p-Rb1 in uninjured and regenerating (2 dpa) *ARF*:GFP fins. Scale bars: 50 μm. Very little p-Rb1 staining is seen in the uninjured fin, but high levels of p-Rb1 staining can be seen in Msxb + cells in the blastema at 2 dpa. The white dashed line represents the amputation plane. (**C**) Representative ChIP qPCR data of three experimental replicates with a pool of 30 fins per experiment. Tissue was collected from *ARF*:GFP transgenic fish before injury (uninj.), at 2 dpa (regenerate only), and at 72 hpf. Fold enrichment of E2f1 binding was normalized to rabbit IgG. The zebrafish thymidine kinase 1 (tk1) promoter was used as a positive control for E2f1 binding. Sequences 2 kbp upstream of tk1 were used as a negative control (tk1-). Values above twofold (black dashed line) are significant (p<0.05). Figure supplement 1 shows promoter sequences for the *ARF, tk1*, and *tk1-* promoters annotated for canonical E2f binding sites. hpa: Hours postamputation. **DOI:**http://dx.doi.org/10.7554/eLife.07702.007

In mammalian cells, *ARF* detects and responds to aberrant inhibition of the Rb pathway (Sharpless, 2005; Sherr, 2006). To investigate the specific factors that activate *ARF* during zebrafish fin regeneration, but not during development, we assessed Rb pathway inhibition by Western blot analysis of E2f1, Rb1, and hyperphosophorylated-Rb1 (p-Rb1) in developing embryos (72 hpf), in adult uninjured fin tissue (uninj.) and at 2 days postamputation (dpa). Whereas p-Rb1 levels were relatively low in uninjured adult fins, a modest increase was detected in 72 hpf embryos. However, 2 dpa regenerates contained a dramatic increase in p-Rb1 levels despite stable levels of total Rb1 and total E2f1 (Figure 2A). This reflects a high level of pro-proliferation signaling resulting in inactivation of Rb1 by phosphorylation, as occurs commonly in tumors. To further investigate where in the regenerating fin the changes in p-Rb1 phosphorylation occurred, immunostaining of regenerating and uninjured fins was performed. Similar to Western blot analysis, immunostaining revealed a dramatic increase in p-Rb1 staining during regeneration (Figure 2B). A small amount of p-Rb1 staining was observed in uninjured fins, which is most likely the result of homeostatic proliferation (Wills et al., 2008). Co-immunostaining for GFP, and Msxb confirmed that p-Rb1 hyperphosphorylation and GFP were co-expressed in cells specifically localized within the blastema.

**Figure 2—figure supplement 1.**
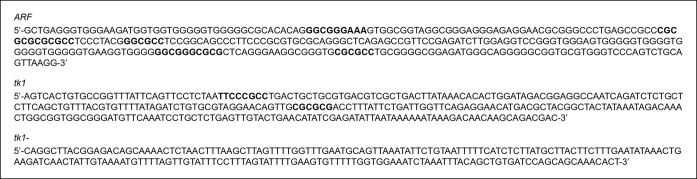
Promoter sequences evaluated for E2f1 enrichment using an E2f1 antibody to perform a ChIP assay. Both *ARF* and *tk1* promoters contain E2f binding sites (bold; del Arroyo et al., 2007, Tfsitescan), but the *tk1-* promoter does not. **DOI:**http://dx.doi.org/10.7554/eLife.07702.008

The hyperphosphorylation of Rb1 in the blastema suggested that resulting elevated levels of free E2f could be sensed by *ARF* resulting in transcriptional activation. Moreover, since E2F can directly activate *ARF* in mammals (Gil and Peters, 2006), we evaluated interaction of fish E2f1 with the mammalian *ARF *promoter by chromatin immunoprecipitation (ChIP) experiments using an anti-E2f1 antibody in developing *ARF*:GFP embryos and uninjured and 2 dpa *ARF*:GFP fins. We analyzed the precipitated DNA fragments by quantitative polymerase chain reaction (qPCR) for three specific genomic regions, the *ARF* promoter, the *tk1* promoter (a known target gene of E2f1; Wells et al., 2002), and a region 2 kilobases (kb) upstream of the *tk1 *promoter (*tk1-*) as a negative control (Figure 2—figure supplement 1). We found that in contrast to the state before amputation, *ARF* is bound by E2f1 specifically during regeneration as is *tk1* but not *tk1-* (Figure 2C). The ChIP assay showed that binding of the *ARF *promoter by E2f1 was enriched over sixfold relative to non-amputated controls and the *tk1-* control. The *ARF* promoter was even more highly enriched than the *tk1+* control. Despite the modestly increased p-Rb1 levels in 72 hpf embryos, which correlated with enrichment of E2f1 at the *tk1* promoter, no increase in E2f1 binding of the *ARF* promoter was observed. This finding suggests that the *ARF *promoter responds strongly and specifically to suprathreshold free E2f1 levels present during regeneration as opposed to other physiological contexts. This result implies that proliferative signaling during fin regeneration has similarities to that during mammalian tumor formation which elicit the *ARF* tumor suppressor response.

### Human ARF suppresses zebrafish fin regeneration

**Figure 3.**
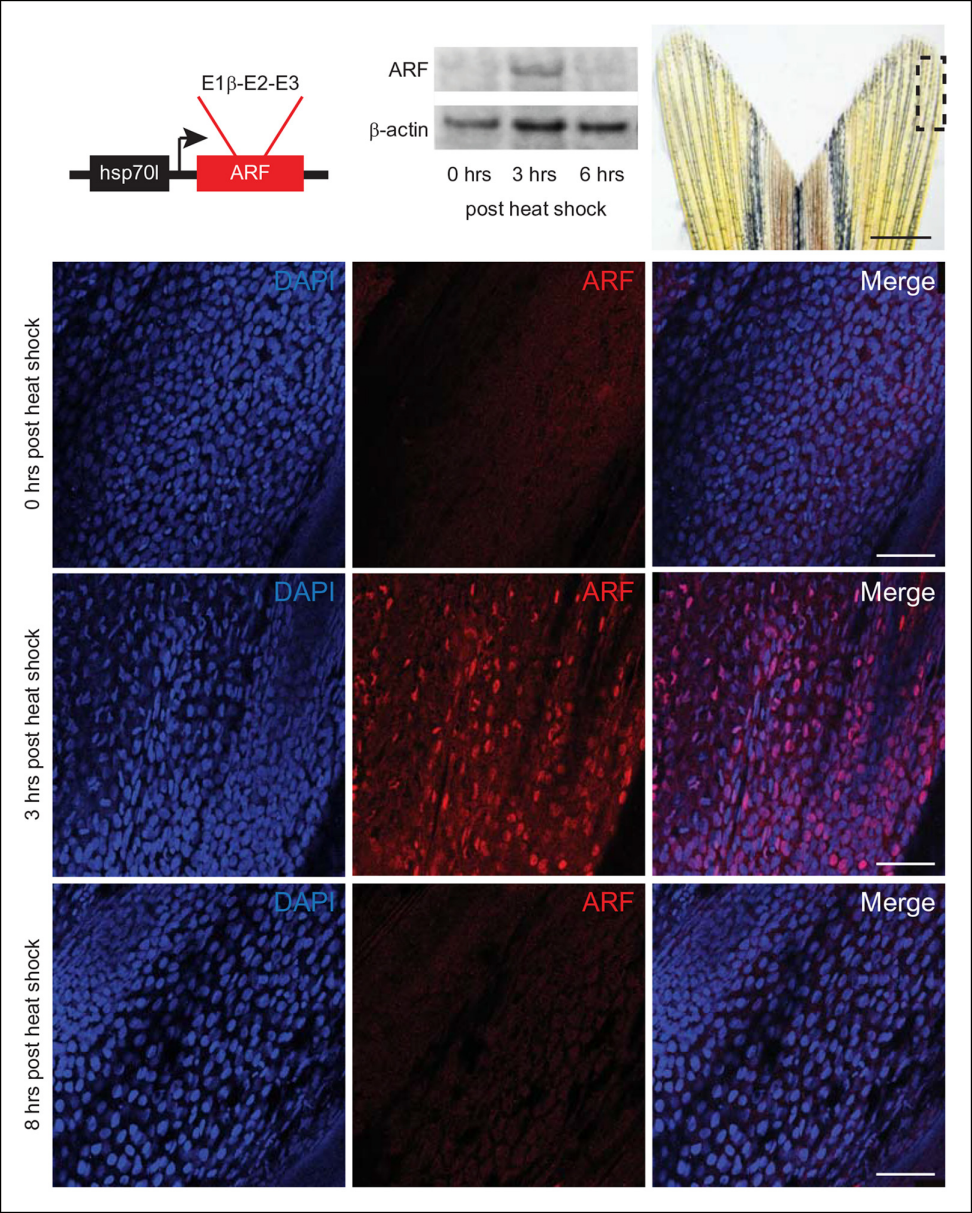
Expression of the mammalian tumor suppressor ARF in zebrafish driven by heat shock promoter. * In vivo* analysis of transgenic zebrafish expressing human ARF under the control of an inducible heat shock promoter, Tg (*hsp70l*:ARF) (*hs*:ARF). Schematic of the *hs*:ARF transgene (top left). The ARF cassette included in the transgene is a cDNA that consists of human exons 1b, 2, and 3 of CDKN2A. Representative Western blot of 3 replicates of ARF before (0 hr) and 3 and 6 hr post heat shock induction of ARF expression (top middle). Portion of fin shown for analysis of expression* in vivo* (top right; dashed box). Scale bar: 1 mm. Immunostaining (sagittal confocal images) for ARF in adult *hs*:ARF zebrafish fins at 0, 3, and 8 hr after a single, hour long, 37°C heat shock (bottom). Scale bars: 50 μm. ARF expression is maximal at 3 hr post heat shock, and it is undetectable by 8 hr post heat shock. Figure supplement 1 shows* in vitro* assays. **DOI:**http://dx.doi.org/10.7554/eLife.07702.009

Since ARF is a human protein with no orthologs in zebrafish, we confirmed the expected subcellular localization of ARF and stabilization of Tp53 in zebrafish cells *in vitro* using the zebrafish cell lines, ZF4 and ZKS (Stachura et al., 2009). Cells were transfected with an ARF expression construct (human ARF cDNA subcloned into pcDNA3) to determine the subcellular localization of the protein as well as to confirm its interactions with orthologs of its mammalian partner, Mdm2 (Sherr, 2006; Sharpless, 2005). Confocal imaging showed that human ARF localized to the nucleolus and co-localized with Mdm2 in zebrafish cells (Figure 3—figure supplement 1A). Tp53 levels were examined in fish cells transfected with ARF expression or control constructs. Elevated Tp53 levels were readily observed in approximately 40% of ARF transfected cells (Figure 3—figure supplement 1A,B). The recapitulation of typical localization and Tp53 upregulation suggested conservation of human ARF functions in zebrafish cells and supported investigation of ARF transgenic fish. To investigate the phenotypic effects of ARF on regeneration *in vivo*, we first utilized the heat shock protein 70 inducible promoter to drive expression of ARF (Tg (*hsp70l*:ARF) or *hs*:ARF) (Figure 3). *hs*:ARF fish were subjected to multiple heat shock regimens to determine ARF expression and stability. Immunostaining showed that when induced with an hour long, 37°C heat shock, ARF is robustly expressed in the fin 3 hr later (Figure 3). Western blot confirmed induction of ARF protein expression and also showed a rapid decrease almost to baseline at 6 hr (Figure 3), in accordance with the known 6 hr half-life of the human protein (Sherr, 2006).

**Figure 3—figure supplement 1.**
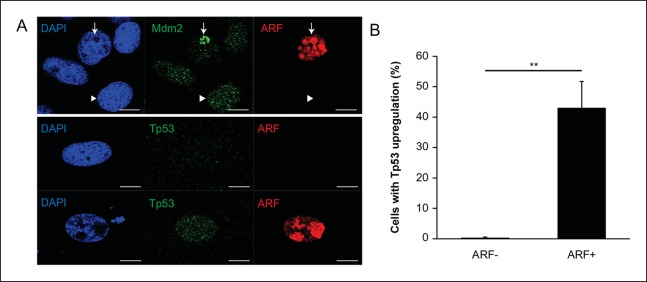
Analysis of ARF expression in zebrafish cells. (**A**) Immunofluorescence for Mdm2 and ARF (top) and Tp53 (bottom) in zebrafish cells (ZKS) transfected with pcDNA-ARF. ARF and Mdm2 co-localize in the nucleolus (arrow) when ARF is expressed; in cells without ARF, Mdm2 has a diffuse nuclear staining pattern (arrow head; top). Tp53 upregulation depends on ARF expression (bottom). Scale bars: 10 μm. (**B**) Quantification of Tp53 upregulation in zebrafish cells (ZKS) transfected with pcDNA-ARF (N = 100, p<0.01). Results are shown as mean ± standard deviation. **DOI:**http://dx.doi.org/10.7554/eLife.07702.010

**Figure 4.**
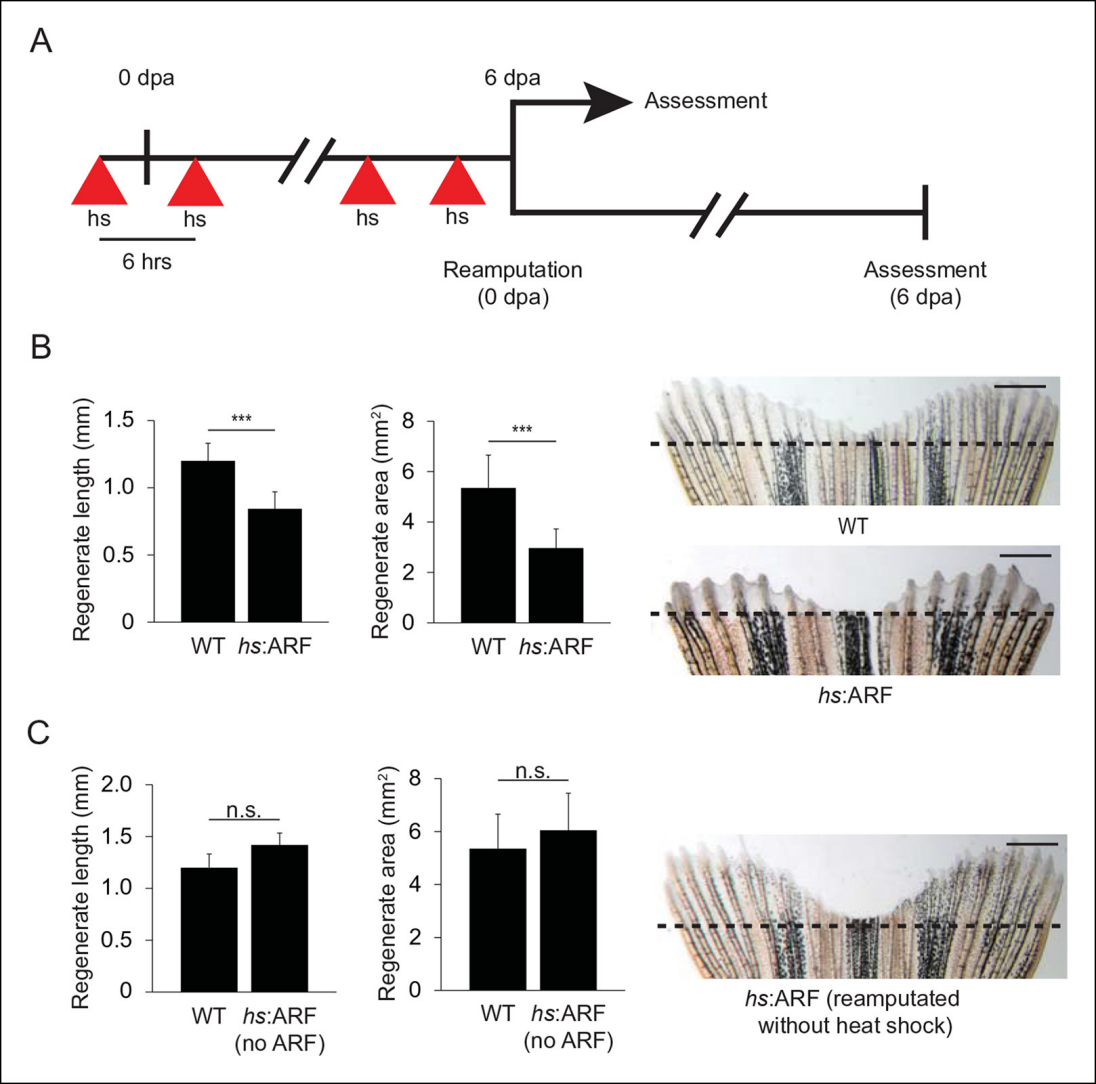
ARF suppresses fin regeneration. (**A**) Schematic of heat shock regimen. An initial hour long, 37°C heat shock is delivered 3 hr prior to amputation (0 dpa) and then every 6 hrs thereafter for 6 days. Regenerates are then assessed (top) or fins are reamputated (0 dpa) and allowed to regenerate in the absence of heat shock for 6 days (bottom). (**B**) Quantification of regenerate length and area at 6 dpa in WT and *hs*:ARF fins exposed to the heat shock regimen (left; N = 40 fins representing multiple different transgene insertions, p<0.001). Representative images of fin regeneration at 6 dpa in WT and *hs*:ARF fins exposed to the heat shock regimen (right). (**C**) Quantification of regenerate length and area at 6 dpa in reamputated *hs*:ARF fins not exposed to heat shock (left; N = 40 fins, p>0.05). Representative image of fin regeneration at 6 dpa in a reamputated *hs*:ARF fin not exposed to heat shock (right). The dashed lines represent amputation planes. Scale bars: 1 mm. Results are shown as mean ± standard deviation. Figure supplement 1 shows ARF and Tp53 immunostaining at 4 dpa, and *tp53* and *cdkn1a* expression changes with ARF expression. It also shows regeneration at 14 dpa after heat shock was discontinued at 6 dpa. hs: Heat shock; WT: Wild type. n.s.: not significant. **DOI:**http://dx.doi.org/10.7554/eLife.07702.011

We then examined the effects of inducible, transient ARF expression on fin regeneration. Using a regimen of one heat shock 3 hr prior to amputation and then subsequently every 6 hrs up to 6 dpa (Figure 4A, top), fin regeneration in *hs*:ARF transgenic fish was compared with non-transgenic wild type (WT) clutchmates. *hs*:ARF and WT fish tolerated heat shock well without overt illness or mortality. ARF expression caused significant inhibition of fin regeneration as evidenced by reduced regenerate length and area; WT regenerates measured 1.2 ± 0.13 mm in length and 5.4 ± 1.3 mm^2^ in area compared with *hs*:ARF regenerates, which measured 0.84 ± 0.13 mm in length and 3.0 ± 0.76 mm^2^ in area, a reduction of approximately 30% (p<0.001) and 45% (p<0.001), respectively (Figure 4B). Inducible ARF expression was confirmed in *hs*:ARF, but not WT fins exposed to heat shock during regeneration (4 dpa) (Figure 4—figure supplement 1A). After the heat shock regimen ended, fin regeneration resumed to reach full length by 14 dpa (Figure 4—figure supplement 1B). Both *hs*:ARF transgenic fish maintained at 28–30°C and WT fish exposed to heat shock regenerated their fins normally. When previously heat-shocked *hs*:ARF fins were reamputated and allowed to regenerate in the absence of heat shock (Figure 4A, bottom), the fins regenerated equally as well as WT fins (Figure 4C). This indicates that ARF inhibits fin regeneration in a reversible manner and that its continued expression is required for regeneration suppression.

**Figure 4—figure supplement 1.**
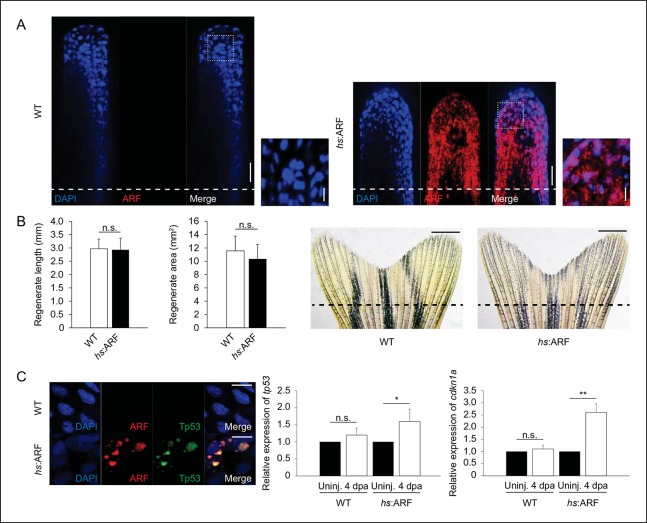
ARF expression during regeneration promotes Tp53, *tp53*, and *cdkn1a* upregulation and regeneration inhibition is reversible. (**A**) Representative (sagittal confocal) images of WT and *hs*:ARF fins at 4 dpa. Scale bars: 50 μm. Dashed lines represent amputation planes. ARF localizes to the nucleus (inset). Scale bars: 10 μm. (**B**) Quantification of fin regenerate length and area in WT and *hs*:ARF at 14 dpa after heat shock was discontinued at 6 dpa (left; N = 10 fins, p>0.05). Representative images of WT and *hs*:ARF fins at 14 dpa after heat shock was discontinued at 6 dpa (right). (**C**) Representative images of ARF and Tp53 in WT and *hs*:ARF fins at 4 dpa (left). Scale bar: 10 μm. Tp53 expression is only detected in cells with ARF expression. Quantifications of relative *tp53* (middle) and *cdkn1a* (right) transcript expression in uninjured (uninj.) WT and *hs*:ARF fin and regenerates at 4 dpa (N = 3 replicates). Expression was normalized to β-Actin transcripts and relative to uninjured fins within each condition. Significant increases in *tp53* (N=5 fins, p<0.05) and *cdkn1a* (N =5 fins, p<0.01) were observed with ARF expression. Results are shown as mean ± standard deviation.hs: Heat shock; WT: Wild type. n.s.: not significant. **DOI:**http://dx.doi.org/10.7554/eLife.07702.012

### ARF suppresses fin regeneration in a p53-dependent manner by inducing apoptosis and causing cell-cycle arrest

**Figure 5.**
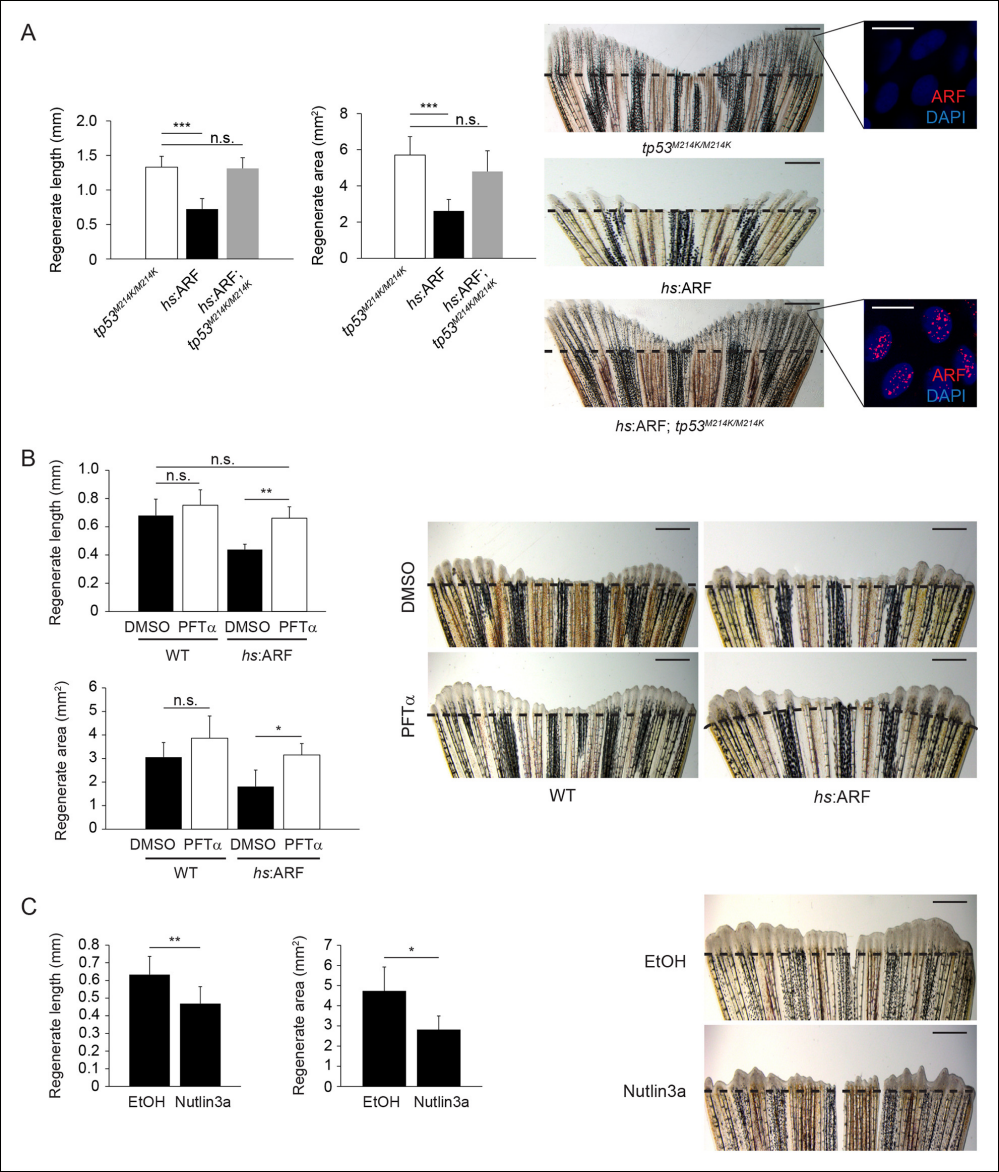
Human ARF functions through the Tp53 pathway in fish to suppress regeneration. (**A**) Quantification of regenerate length and area at 6 dpa in *tp53^M214K/M214K^, hs*:ARF, and *hs*:ARF; *tp53^M214K/M214K^* fins exposed to the heat shock regimen as in Figure 4 (left; N = 30 fins). Representative images of fin regeneration at 6 dpa in *tp53^M214K/M214K^, hs*:ARF, and *hs*:ARF; *tp53^M214K/M214K^* fins exposed to heat shock (right). Scale bars: 1 mm. Immunostaining (sagittal confocal images) for ARF in *tp53^M214K/M214K^* and *hs*:ARF; *tp53^M214K/M214K^* fins 3 hr after a single heat shock (right inset). Scale bars: 10 μm. Fin regeneration proceeds equally well in *tp53^M214K/M214K^* and *hs*:ARF; *tp53^M214K/M214K^* fins exposed to heat shock, but fin regeneration inhibition is observed in *hs*:ARF fins exposed to heat shock. (**B**) Quantification of regenerate length and area at 4 dpa in wild type (WT) and *hs*:ARF fins exposed to heat shock and 5 μM pifithrin-α(PFTα) or 0.1% Dimethyl sulfoxide (DMSO) (vehicle) (left; N = 8 fins, p<0.01). Representative images of fin regeneration at 4 dpa in WT and *hs*:ARF fins exposed to heat shock and 5 μM PFTα or 0.1% DMSO (right). Scale bars: 0.5 mm. Inhibition of Tp53 activity with PFTα rescues regeneration suppression by ARF. (**C**) Quantification of regenerate length and area at 4 dpa in WT fins exposed to 5 μM nutlin3a or Ethanol (EtOH) (vehicle) (left; N = 8 fins, p<0.01). Representative images of fin regeneration at 4 dpa in WT fins exposed to 5 μM nutlin3a or EtOH (right). Scale bars: 0.5 mm. Inhibition of Mdm2 with nutlin3a phenocopies ARF expression by suppressing fin regeneration. The dashed lines represent amputation planes. Results are shown as mean ± standard deviation.  n.s.: not significant. **DOI:**http://dx.doi.org/10.7554/eLife.07702.013

To assess whether ARF functions through the p53 pathway to inhibit fin regeneration**in vivo, we examined Tp53 protein and transcript levels as well as induction of the p53 target gene *cdkn1a *(p21) in response to ARF expression at 4 dpa (Figure 4—figure supplement 1C). The induction and stabilization of p53 and induction of *cdkn1a* transcripts by ARF showed that ARF impacts p53 functions *in vivo* in fish regenerates. To assess the Tp53-dependence of ARF, we first crossed *hs*:ARF fish with *tp53^M214K^* mutant fish to generate *hs*:ARF fish that are homozygous for the tp53^M214K^ allele (*hs*:ARF; *tp53^M214K/M214K^*). The tp53^M214K^ mutation abrogates Tp53 transactivation functions (Berghmans et al., 2005). Using the same amputation and heat shock regimen, we analyzed fin regeneration and found no difference in regenerate length or area despite ARF expression in *tp53* mutant fish (Figure 5A). We also tested Tp53-dependence of ARF regeneration suppression by treating zebrafish with either pifithrin-α (PFTαSigma, St. Louis, MO), an inhibitor of Tp53 transactivation (Komarov et al., 1999), or nutlin3a, a molecule that disrupts the Mdm2–Tp53 interaction, thereby stabilizing Tp53 levels (Yun et al., 2013; Vassilev et al., 2004). Treatment of *hs*:ARF and WT fish with 5 μM PFTα and heat shock increased *hs*:ARF regenerate length from 0.44 ± 0.04 mm to 0.66 ± 0.08 mm, an increase of 50% (p<0.01), and area from 1.8 ± 0.7 mm^2^ to 3.2 ± 0.5 mm^2^, an increase of approximately 75% (p<0.05), compared with carrier-treated controls. Fin regeneration of WT fish was not affected by PFTα treatments (Figure 5B). Treatment of WT fish with 5 μM nutlin3a reduced fin regenerate length from approximately 0.65 ± 0.1 mm to 0.45 ± 0.1 mm, a decrease of 30% (p<0.01), and area from 4.7 ± 1.2 mm^2^ to 2.8 ± 0.7 mm^2^, a reduction of 40% (p<0.05) (Figure 5C, left), phenocopying the fin regeneration inhibition phenotype of induced *hs*:ARF fish (Figure 5C, right). Together, these experiments show that ARF functions through Tp53-dependent mechanisms to inhibit fin regeneration, and also demonstrate the importance of active suppression of Tp53 by Mdm2.

**Figure 6.**
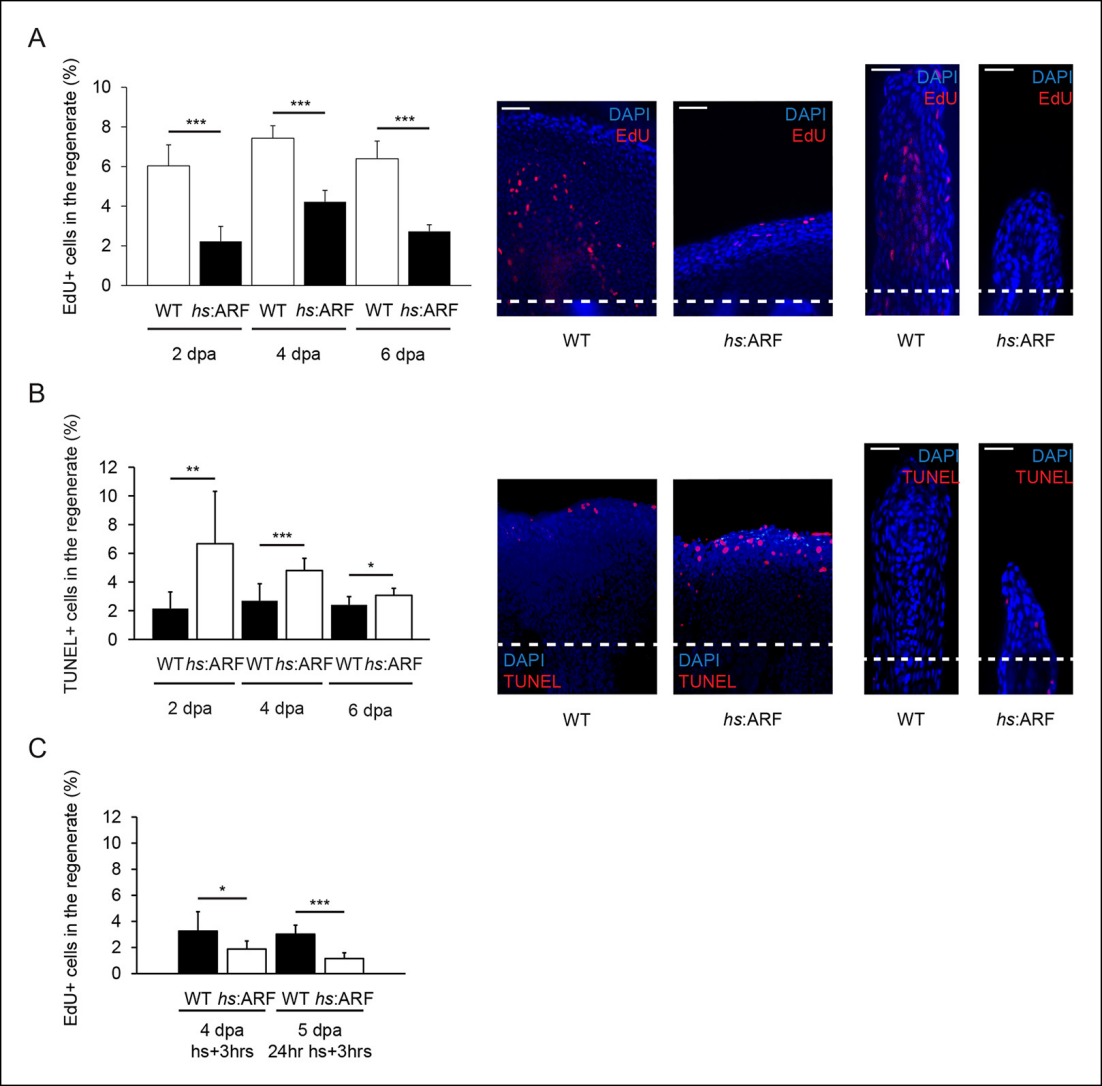
ARF suppresses fin regeneration by inducing apoptosis and cell-cycle arrest. (**A**) Quantification of EdU staining at 2, 4, and 6 dpa in wild type (WT) and *hs*:ARF fins exposed to heat shock (left). At 2 dpa, 6.0% ± 1.1% of cells in WT regenerates were EdU + compared with approximately 2.2% ± 0.8% in Heat shock (*hs*):ARF regenerates. At 4 dpa, approximately 7.4% ± 0.6% of cells in WT regenerates were EdU + compared with 4.2% ± 0.6% in *hs*:ARF regenerates. At 6 dpa, approximately 6.4% ± 0.9% of cells in WT regenerates were EdU + compared with 2.7% ± 0.3% in *hs*:ARF regenerates. Significantly fewer cycling cells are detected with ARF expression (N = 10 fins, p<0.001). Representative (left – sagittal confocal, right – longitudinal) images of EdU staining at 2 dpa in WT and hs:ARF fins exposed to heat shock (right). Scale bars: left – 50 μm, right – 25 μm. Dashed lines represent amputation planes. (**B**) Quantification of Terminal deoxynucleotidyl transferase dUTP nick end labeling (TUNEL) staining at 2, 4, and 6 dpa in WT and *hs*:ARF fins exposed to heat shock (left). At 2 dpa, 2.2% ± 1.2% of cells in WT regenerates were TUNEL + , while 6.7% ± 3.7% of cells in *hs*:ARF regenerates were TUNEL + . At 4 dpa, only 2.7% ± 1.2% of cells in WT regenerates were TUNEL + compared with 4.8% ± 0.8% in *hs*:ARF regenerates. At 6 dpa, 2.4% ± 0.6% of cells in WT regenerates were TUNEL +, while 3.1% ± 0.5% of cell in *hs*:ARF regenerates were TUNEL +. Significantly more apoptosis is detected with ARF expression (N = 10 fins, p<0.001). Representative images (left – sagittal, right – longitudinal) of TUNEL staining at 2 dpa in WT and *hs*:ARF fins exposed to heat shock (right). Image quantification was performed on regenerates only. Dashed lines represent amputation planes. Scale bars: left – 50 μm, right – 25 μm. (**C**) Quantification of EdU staining in WT and *hs*:ARF fins 3 hr after a single heat shock or 24 hr of heat shock delivered at 4 dpa. After a single heat shock, 3.3% ± 1.5% of cells in WT regenerates were EdU + compared with 1.9% ± 0.6% in *hs*:ARF regenerates. After 24 hr of heat shock, 3.0% ± 0.7% of cells in WT regenerates were EdU + compared with 1.2% ± 0.4% in *hs*:ARF regenerates. Significantly fewer cycling cells are detected with ARF expression after blastema formation (N = 10 fins, p<0.001). Results are shown as mean ± standard deviation. **DOI:**http://dx.doi.org/10.7554/eLife.07702.014

In order to understand the cellular effects of ARF that lead to inhibition of fin regeneration, we examined apoptosis and proliferation in blastema cells during regeneration with and without ARF expression. To estimate cell proliferation differences between WT and *hs*:ARF fins, EdU pulse-chase experiments were performed at 2, 4, and 6 dpa. EdU incorporation was significantly higher in WT fin regenerates compared with *hs*:ARF regenerates at all time points examined with the greatest difference occurring at 2 dpa (171%, p<0.001) (Figure 6A). Apoptotic cells in *hs*:ARF and WT regenerates were analyzed using terminal deoxynucleotidyl transferase dUTP nick end labeling (TUNEL) staining. When the percent of TUNEL + cells in WT and *hs*:ARF regenerates was compared, the incidence of apoptosis increased with ARF expression at all time points examined with the greatest difference occurring at 2 dpa (210%, p<0.01) (Figure 6B). To assess whether ARF directly affects proliferating blastema cells, we also measured EdU incorporation with different heat shock regimens starting at 4 dpa. The results showed that either a single heat shock or 24 hr of heat shocks at 4 dpa significantly reduced the number of cycling cells in the regenerate, demonstrating a direct effect of ARF on the regenerating cell population (Figure 6C).

### *ARF* does not affect development but suppresses fin regeneration in response to regeneration signals

**Figure 7.**
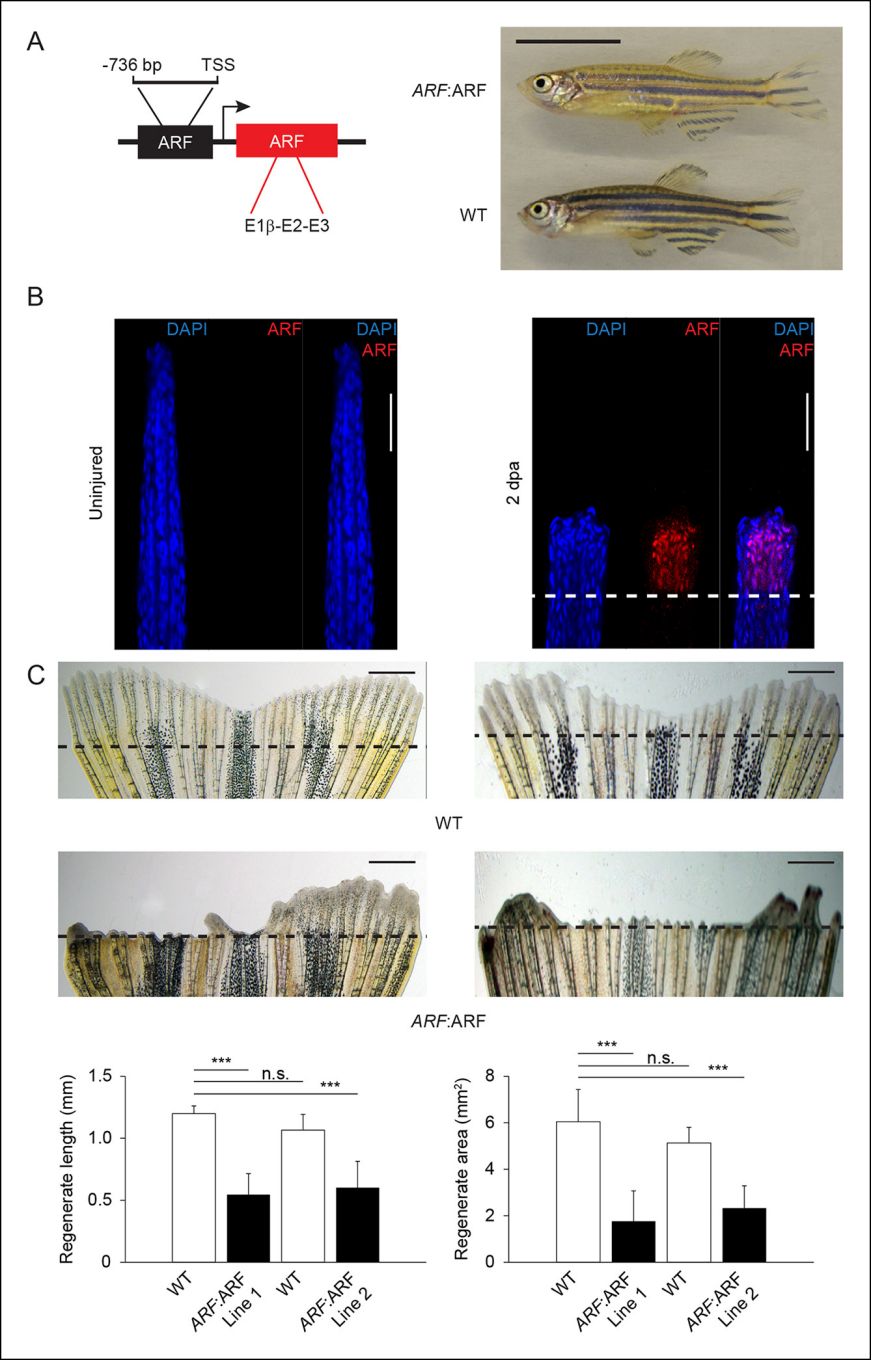
ARF senses regenerative signals and suppresses fin regeneration. (**A**) Schematic of transgene expressing human ARF under the control of the human *ARF* promoter (left). Representative images of age- and sex-matched *ARF*:ARF and WT zebrafish (right; 5 months postfertilization, male). Scale bar: 1 cm. *ARF*:ARF fish are viable, grow to adulthood and are of normal size and patterning. (**B**) Immunostaining (longitudinal confocal images) for ARF in *ARF*:ARF transgenic fish before injury (uninjured ) and at 2 dpa. Scale bars: 50 μm. ARF is specifically expressed upon injury. The dashed line represents the amputation plane. (**C**) Representative images of fin regeneration at 6 dpa in WT and *ARF*:ARF fins (top). Scale bars: 1 mm. The dashed lines represent amputation planes. Quantification of regenerate length and area at 6 dpa in WT and *ARF*:ARF fins (bottom; N= 10 fins, p<0.001). The first set of bars in each graph represents the results from one transgenic line (Line 1), while the second set of bars represents the results from a second, independent transgenic line (Line 2). *ARF* causes marked inhibition of fin regeneration. Results are shown as mean ± standard deviation. Figure supplement 1 shows the embryonic viability of ARF transgenic lines. Figure supplement 2 shows the failure of *ARF*:ARF fins to completely regenerate after 15 days and even 30 days. Figure supplement 3 shows ARF immunostaining at 6 dpa, Tp53, *tp53*, and *cdkn1a* expression changes with ARF expression in WT and *ARF*:ARF fins at 4 dpa, fin regeneration rescue in *ARF*:ARF fins treated with PFTα, and EdU incorporation studies performed in WT and *ARF*:ARF fins. TSS: Transcriptional state site; uninj.: Uninjured; WT: Wild type. n.s.: not significant. **DOI:**http://dx.doi.org/10.7554/eLife.07702.015

Since the *ARF *promoter is activated specifically in the fin during regeneration, we tested how transgenic fish expressing ARF under control of the endogenous *ARF *promoter would develop and regenerate. To do so, we generated zebrafish lines from independent transgenic insertions that utilize the human *ARF* promoter to drive ARF expression (Tg (*ARF*:ARF) or *ARF*:ARF) (Figure 7A, left). ARF expression during development would be expected to adversely affect *ARF*:ARF fish. We observed, however, that *ARF*:ARF transgenic fish are viable, develop normally, and have no overt size or morphological differences when compared with age- and sex-matched WT counterparts (Figure 7A, right). Furthermore, examination of *ARF*:ARF embryos confirmed our findings in *ARF*:GFP transgenics. In agreement with the predictions of *ARF*:GFP experiments, there was no effect of the *ARF*:ARF transgene on survival during early embryogenesis compared with WT fish (Figure 7—figure supplement 1A). We also did not detect ARF expression in embryos, as expected given our findings with *ARF*:GFP fish (Figure 7—figure supplement 1A). To assess whether ARF, if expressed, would interfere with organogenesis or development, we evaluated the effects of induced ARF expression using *hs*:ARF fish. Upon heat shock, *hs*:ARF, but not WT clutches, exhibited drastically reduced survival that was associated with high levels of ARF expression throughout the embryo (Figure 7—figure supplement 1B). This finding indicates that ARF expression is very poorly tolerated by developing embryos and clearly implies that in *ARF*:ARF fish, ARF is not activated significantly during development to affect normal developmental growth and organogenesis.

**Figure 7—figure supplement 1.**
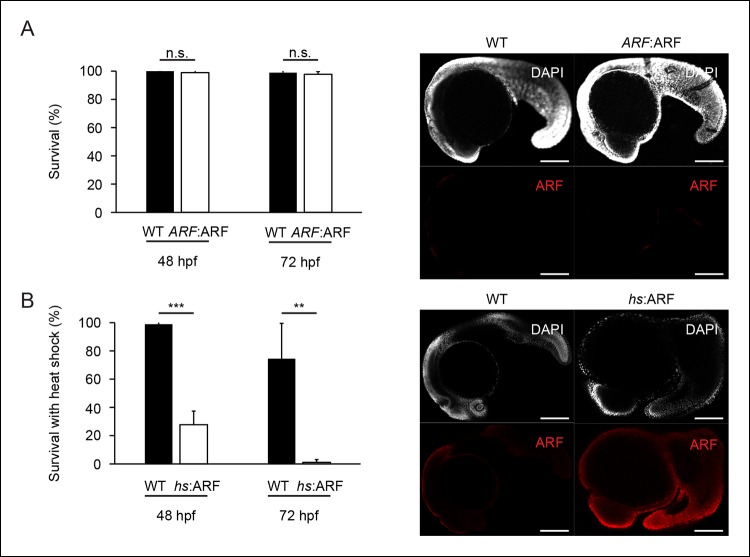
The *ARF*:ARF transgene does not interfere with development, whereas forced ARF expression causes embryonic lethality. (**A**) Quantification of embryonic mortality at 48 hpf and 72 hpf in wild type (WT) and *ARF*:ARF embryos (left; N = 90, p>0.05). Representative sagittal confocal images of ARF expression at 24 hpf in WT and *ARF*:ARF (right). (**B**) Quantification of embryonic mortality at 48 hpf and 72 hpf in WT and *hs*:ARF embryos exposed to heat shock (left; N = 90, p<0.001). Representative sagittal confocal images of ARF expression at 27 hpf in WT and *hs*:ARF embryos 3 hr after a single heat shock (right). Scale bars: 200 μm. Results are shown as mean ± standard deviation . n.s.: not significant. **DOI:**http://dx.doi.org/10.7554/eLife.07702.016

**Figure 7—figure supplement 2.**
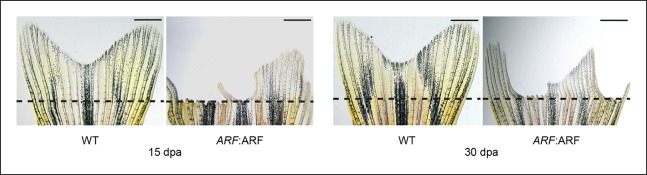
*ARF*:ARF fins do not completely regenerate even after 30 days. Representative images of fin regeneration at 15 dpa and 30 dpa in wild type (WT) and *ARF*:ARF fins. Scale bars: 1 mm. **DOI:**http://dx.doi.org/10.7554/eLife.07702.017

**Figure 7—figure supplement 3.**
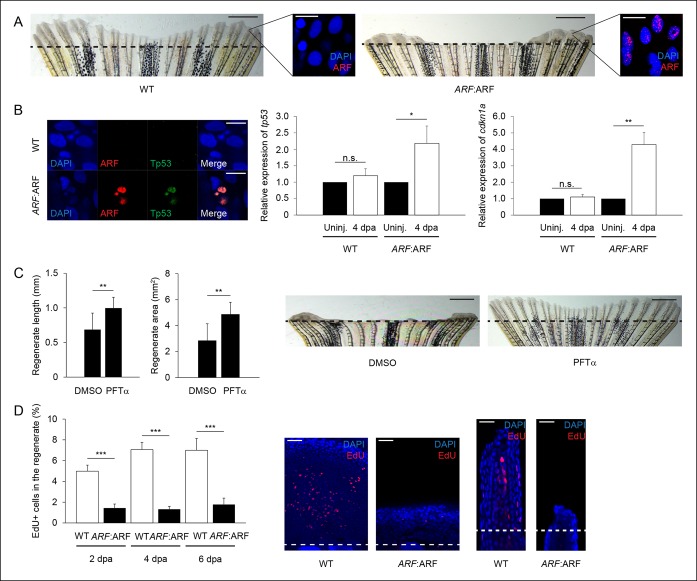
*ARF*:ARF expression and p53-dependent functions during regeneration. (**A**) Representative images of wild type (WT) (left) and *ARF*:ARF (right) fins at 6 dpa. Scale bars: 1 mm. Representative images of ARF expression at 6 dpa in WT (left) and *ARF*:ARF (right) fins. Scale bars: 10 μm. Dashed lines represent amputation planes. (**B**) Representative images of ARF and Tp53 in WT and *ARF*:ARF fins at 4 dpa (left). Scale bar: 10 μm. Tp53 expression is only detected in cells that express ARF. Quantification of relative *tp53* (middle) and *cdkn1a* (right) transcript expression in uninjured (uninj.) WT and *ARF*:ARF fin and regenerates at 4 dpa (N = 3 replicates). Expression was normalized to β-Actin transcripts and relative to fins within each condition. Significant increases in *tp53* (N = 5 fins, p<0.05) and *cdkn1a* (N = 5 fins, p<0.01) were observed with ARF expression. (**C**) Quantification of regenerate length and area at 6 dpa in *ARF*:ARF fins treated with 0.1% Dimethyl sulfoxide (DMSO) or 5 μM Pifithrin-α (PFTα) (left; N = 8 fins/condition, p<0.01). Representative images of fin regeneration at 6 dpa in *ARF*:ARF fins treated with 0.1% DMSO or 5 μM PFTα (right). Scale bars: 1 mm. Dashed lines represent amputation planes. Treatment with PFTα rescues fin regeneration in ARF:ARF transgenic zebrafish. (**D**) Quantification of EdU staining at 2, 4, and 6 dpa in WT and *ARF*:ARF fins (left). At 2 dpa, 5.0% ± 0.6% of cells in WT regenerates were EdU + compared with approximately 1.4% ± 0.4% in *hs*:ARF regenerates. At 4 dpa, approximately 7.0% ± 0.7% of cells in WT regenerates were EdU + compared with 1.3% ± 0.3% in *hs*:ARF regenerates. At 6 dpa, approximately 7.0% ± 1.1% of cells in WT regenerates were EdU + compared with 1.8% ± 0.6% in *hs*:ARF regenerates. Significantly fewer cycling cells are detected with ARF expression (N = 10 fins, p<0.001). Representative (left – sagittal confocal, right – longitudinal) images of EdU staining at 2 dpa in WT and *ARF*:ARF fins (right). Scale bars: left – 50 μm, right – 25 μm. Dashed lines represent amputation planes. Results are shown as mean ± standard deviation. n.s.: not significant. **DOI:**http://dx.doi.org/10.7554/eLife.07702.018

We then performed fin regeneration experiments with *ARF*:ARF transgenic fish and WT fish. When *ARF*:ARF fins were injured, ARF was detected in the regenerate (Figure 7B), and the pattern of expression was similar to GFP expression in *ARF*:GFP regenerates at the same time point (Figure 1). When fin regeneration was compared between *ARF*:ARF transgenic fish and WT fish, *ARF*:ARF regenerates measured 0.55 ± 0.17 mm in length and 1.8 ± 1.3 mm^2^ in area, while WT regenerates measured 1.2 ± 0.06 mm in length and 6.0 ± 1.4 mm^2^ in area. To rule out position effects of the transgene insertion, a second independent transgenic *ARF*:ARF line was also assessed. Regenerates of this second *ARF*:ARF line measured 0.6 ± 0.2 mm in length and 2.3 ± 0.96 mm^2^ in area, while WT regenerates measured 1.1 ± 0.13 mm in length and 5.1 ± 0.67 mm^2^ in area. In all, *ARF*:ARF regenerates were 55% (p<0.001) and 44% (p<0.001) shorter and 70% (p<0.001) and 55% (p<0.001) smaller in area than WT regenerates, and anatomical fin defects persisted 1 month after amputation (Figure 7C, Figure 7—figure supplement 2). ARF expression persisted in *ARF*:ARF fins but not WT fins at 6 dpa (Figure 7—figure supplement 3A), a time point at which GFP in no longer observed in regenerated *ARF*:GFP fins, suggesting ongoing regeneration attempts in *ARF*:ARF fins.

We confirmed that fin regeneration inhibition in *ARF*:ARF fish was p53 dependent as in *hs*:ARF fish. Tp53, *tp53*, and *cdkn1a* expression increased with ARF expression in *ARF*:ARF fins at 4 dpa (Figure 7—figure supplement 3B). As in *hs*:ARF fish, treatment of ARF:ARF fins with 5 μM PFTα rescued fin regeneration (Figure 7—figure supplement 3C). Finally, we quantified the cell cycle arrest that is a consequence of ARF expression comparing WT and *ARF*:ARF fins with EdU pulse-chase labeling at 2, 4, and 6 dpa. Similar to *hs*:ARF fins, *ARF*:ARF fin regenerates had significantly fewer proliferating cells than WT fin regenerates at all time point assessed with the largest difference observed at 4 dpa (81%, p<0.001) (Figure 7—figure supplement 3D).

These results confirm that *ARF* activation is specific to regenerating tissue and show that the magnitude of activation is sufficient to inhibit regeneration. Thus, the presence of a functional human *ARF *gene in fish results in a diminished regenerative capacity, including absence of epimorphic regeneration, without significantly affecting other major physiological or developmental characteristics.

## Discussion

In this study, we have experimentally tested the hypothesis that tumor suppressor evolution may impact regenerative capacity. We found that the core mammalian tumor suppressor *ARF* senses regeneration signals and specifically responds to negatively alter the proliferative balance in the zebrafish blastema, greatly perturbing regeneration. Our findings provide the first *in vivo* experimental evidence that evolution of tumor suppressors can negatively impact solid tissue regeneration potential.

Although the core tumor suppressors as a whole support regenerative processes, the properties of *ARF* identified in this study are at odds with epimorphic regeneration. This new example of antagonistic pleiotropy adds to previously recognized trade-off characteristics of tumor suppressor genes affecting mammalian stem cell function (Pardal et al., 2005; Greaves, 2007; Pomerantz and Blau, 2013; Rodier et al., 2007) and shows that *ARF* antagonistic properties also manifest in the context of the blastema. The evidence that ARF is a critical tumor suppressor in mammals (Sherr, 2006, Sharpless, 2005), but opposes regeneration functions (Sharpless and DePinho, 2007), suggests that the selective pressure that has driven the evolution of *ARF* has primarily enhanced tumor suppression either at the expense of or in the absence of regeneration pressures. Although our experiments and those of others (Gemberling et al., 2013; Poss et al., 2003) show that the regenerative capacity of zebrafish is vulnerable to single gene alterations, whether altering function of a single gene in mammals would induce the emergence of robust epimorphic regenerative capacity is a much more complex issue. Indeed, the multifactorial genetic differences of highly and less regenerative vertebrates make it unlikely that manipulation of a single gene could enable regeneration. It is notable, however, that *Cdkn1a (p21*) knockout mice do possess a somewhat enhanced ability to regenerate solid structures (Heber-Katz et al., 2013; Clark et al., 1998) such as pinnae, which lack the complex tissue structure of a digit, but nonetheless, demonstrate that alteration of cellular growth control mechanisms can impact regeneration. Moreover, the importance of active repression of *ARF* to maintain stem cell function (Molofsky et al., 2005), and of *ARF* reduction to facilitate dedifferentiation (Pajcini et al., 2010) have been documented.

Among the core tumor suppressor genes that are frequently inactivated in mammalian tumors, *ARF *is unique in that it does not have orthologs represented in most vertebrates including highly regenerative species. By contrast, *Tp53, Pten, *and* Ink4a *have distant orthologs, present in invertebrates and vertebrates alike. The transgenesis approach we used to study ARF in fin regeneration made it possible for us to study ARF with its human regulatory components but without increasing *CDKN2A* CKI gene dosage, which could have been a complicating factor in a transgenic harboring the entire *CDKN2A (INK4A/ARF)* locus. This study extends our previous observations (Pajcini et al., 2010) that ARF prevents dedifferentiation in muscle cells in culture and provides new evidence that ARF functions *in vivo* to oppose tissue regeneration. Future experiments will determine whether ARF prevents dedifferentiation *in vivo*, such as the dedifferentiation of osteoblasts in regenerating fins, or whether it acts on proliferating blastema cells after they have dedifferentiated. Combined, our findings suggest that zebrafish cells are more promiscuous in terms of tolerance to high levels of mitogenic activity, thus permitting the cellular processes required for epimorphic regeneration. It follows that regenerating cells in organisms that have an *ARF* gene would need to prevent ARF activation or would be inherently more restricted in these activities.

**Figure 8.**
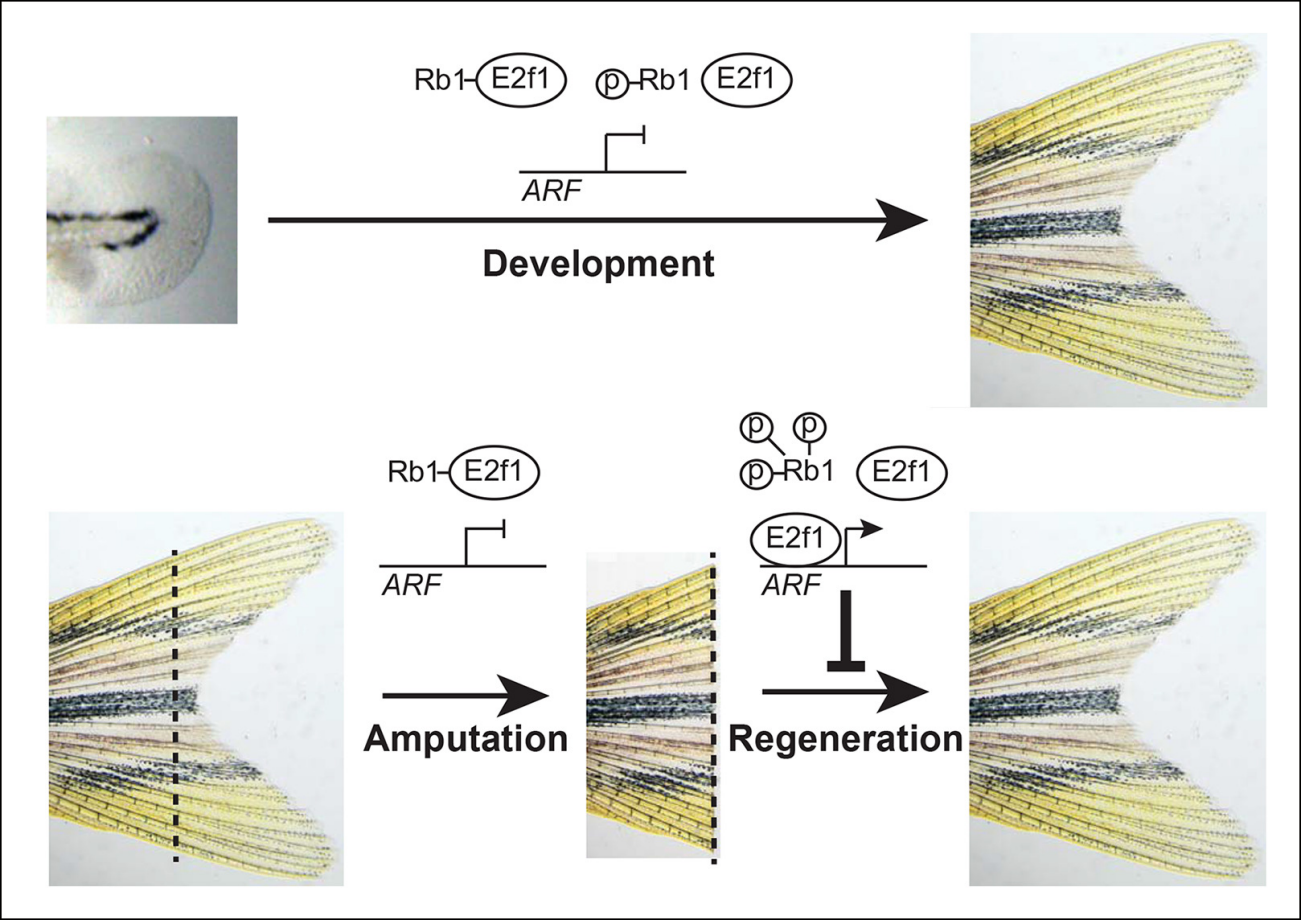
Model of *ARF* function in the context of Rb pathway activity during zebrafish development and fin regeneration. *ARF* is not active during development during which a moderate level of mitogenic signaling causes modest phosphorylation of Rb1 (top); however, during regeneration, high mitogenic signaling induces Rb1 hyperphosphorylation and abundant free E2f1, which activates *ARF* and leads to inhibition of regeneration (bottom). The dashed lines represent the amputation plane. **DOI:**http://dx.doi.org/10.7554/eLife.07702.019

We found that ARF recapitulates its core mammalian mechanistic functions in zebrafish cells and tissues. As in mammals, when ARF is overexpressed in zebrafish cells, it associates with Mdm2, stabilizes Tp53, and induces cell cycle arrest or apoptosis. This functional conservation over an evolutionary distance demonstrates that cross-species genetic variations can be experimentally examined in the study of regeneration. When ARF expression is driven by its endogenous human promoter in zebrafish cells, activation of the p53 axis occurs specifically in the blastema-regeneration scenario. Remarkably, the inhibitory effect on regeneration by *ARF*:ARF was stronger than with the heat shock promoter, probably reflecting ongoing surveillance of regenerative signals by the *ARF* promoter in contrast to fluctuating ARF levels obtained with intermittent heat shock induction of a short half-life protein. In the developing or adult uninjured state, E2f1 is sequestered and inhibited by Rb1, and *ARF* is inactive. However, during blastema formation and regeneration, Rb1 hyperphosphorylation is associated with sufficient free E2f1 to activate *ARF*, which inhibits fin regeneration via a Tp53-dependent mechanism (Figure 8). Our findings and model are in agreement with the recent proposal that in salamanders the absence of ARF permits downregulation of Tp53 during blastema formation (Yun et al., 2013). The responsiveness of *ARF* to the Rb pathway proliferative signaling characteristic of zebrafish fin regeneration implies that similar mitogenic signaling occurring in a mammalian context would be detected as aberrant, activate ARF–MDM2–TP53 tumor suppressor mechanisms, and oppose regeneration. Our findings are compatible with previous mouse studies showing that ARF is a potent tumor suppressor that is dispensable for normal development (Serrano et al., 1996; Kamijo et al., 1997). Moreover, prior observations that *ARF* is not developmentally expressed in the majority of tissues in the mouse (Gromley et al., 2009; Zindy et al., 2003) support the fidelity of the promoter used in this study. Although the majority of tumor suppressors probably function in regeneration as they do in normal development, the findings of the present study indicate that *ARF* represents an unusual departure from that paradigm in that the properties that cause it to respond specifically to tumorigenesis also cause it to distinguish regeneration contexts from developmental ones.

We show here how examination of zebrafish that are humanized with respect to candidate regeneration modifiers is informative for understanding disparate regenerative capacities. Such an approach should prove useful for examining other candidate genes and pathways of interest. Our findings with respect to ARF strongly suggest that it is a barrier to mammalian epimorphic regeneration because it interprets the regeneration context as similar to tumorigenesis. It follows conceptually that approaches to induce epimorphic regeneration clinically would need to disrupt ARF–MDM2–TP53 axis activation.

## Materials and methods

### Zebrafish

Zebrafish maintenance at 28–30°C and all experiments were approved by the Institutional Animal Care and Use Committee of the University of California, San Francisco. Three- to six-month-old WT or transgenic AB zebrafish were used for all experiments. The Tg (hsp70l:ARF), Tg (ARF:ARF), and Tg (ARF:GFP) constructs were created by either subcloning the cDNA of human ARF (exons 1β, 2, and 3 of *CDKN2A*) or a cytoplasmic EGFP cassette downstream of either the promoter sequences of zebrafish *hsp70l* (Halloran et al., 2000) or the human *CDKN2A* promoter (Robertson and Jones, 1998), respectively. The *ARF* promoter was subcloned from pKR19 (Robertson and Jones, 1998) using SalI to excise an approximately 1 kb region of the human promoter, which encompassed 736 bp 5^′^ of the transcriptional state site of *ARF*. Sequence information can be found in del Arroyo et al. (2007). Tol2-mediated transgenesis was used to generate transgenic animals (Kwan et al., 2007). Transgenic animals were detected based on their GFP-positive hearts, due to the transgenes containing a *cmlc2*:GFP cassette. All transgenic strains were analyzed as hemizygotes. For drug treatment experiments, zebrafish were treated with 5 μM αPTFα in dimethyl sulfoxide (DMSO) (5 mM stock) or 5 μM (-)-Nutlin-3 (Cayman, Ann Arbor, MI) in ethanol (EtOH) (5 mM stock). Water was exchanged daily. For EdU pulse-chase experiments, 5 μL of 5 mg/mL of EdU (Life Technologies, Carlsbad, CA) in saline was injected intraperitoneally into anesthetized fish 30 min before tissue harvest.

### Immunostaining

Zebrafish fin immunostaining was performed on whole-mounted fins as previously described (Sousa et al., 2011). For coronal views, whole-mount stained fins were embedded in 5% agarose, and 200 μm sections were cut with a vibratome. Imaging was performed with a confocal microscope. Zebrafish embryo immunostaining was performed on whole-mounted, 1-phenyl 2-thiourea (PTU; Sigma)-treated embryos as previously described (Macdonald, 1999). Zebrafish cell immunostaining: 4% paraformaldehyde (PFA) 10 min, phosphate-buffered saline (PBS) 5 min 3×, 0.3% PBTx 15 min, PBS 5 min 3×, serum-free protein block (Dako, Carpinteria, CA) 1 hr, primary antibodies in antibody diluent (Dako) overnight 4°C, PBS 5 min 3×, secondary antibodies in antibody diluent 1 hr, PBS 5 min 3×, mounted with Vectashield mounting medium with DAPI (Vector Laboratories, Burlingame, CA). EdU incorporation was detected using the Click-iT EdU Imaging Kit per the manufacturer’s instructions (Life Technologies). TUNEL detection was performed using the In Situ Cell Death Detection Kit (Roche, Basel, Switzerland) per the manufacturer’s instructions. Images were quantified in ImageJ. The percent of EdU + or TUNEL + cells was quantified by first counting the number of positive cells in the regenerate and then dividing that count by the number of nuclei in the field counted.

### *In situ* hybridization

Zebrafish embryo mRNA *in situ* hybridization was performed on whole-mounted, PTU-treated embryos as previously described (Chitramuthu and Bennett, 2013). The antisense GFP probe was labeled with digoxigenin-11-UTP (Roche) and generated using the following primers: 5′-AAGGGCGAGGAGCTGTTCAC-3^′^ and 5^′^-GAACTCCAGCAGGACCATGT-3^′^ (MacDonald et al., 2010).

### Western blot

An amount of 50–60 μg of total protein isolated from adult zebrafish fin tissue was loaded per lane, electrophoresed, and transferred to polyvinyl difluoride (PVDF) membranes. Protein was visualized using ECL Prime (GE Healthcare Bio-Sciences, Pittsburgh, PA) and an ImageQuant LAS 4000 (GE Healthcare Bio-Sciences). Band quantification was performed using ImageQuantTL software. For each condition, Rb1 and p-Rb1 bands were normalized to β-Actin, and the ratio of p-Rb1:Rb1 was calculated and made relative to uninjured tissue (Table 1).

**Table 1.** Primary antibodies. **DOI:**http://dx.doi.org/10.7554/eLife.07702.020

Host species	Antigen	Company	Cat. No.	Dilution	Application
Mouse	Tp53	Abcam	ab77813	1:50	IHC
Rabbit	Mdm2	Santa Cruz	C-18	1:50	IHC
Rabbit	GFP	Torrey Pines	TP401	1:3000	IHC
Chicken	GFP	Abcam	ab13970	1:3000	IHC
Mouse	Msxb	DSHC	4G1-c	1:50	IHC
Rabbit	PCNA	Abcam	ab2426	1:500	IHC
Mouse	p14ARF	Cell Signaling	2407	1:100/1:500	IHC/WB
Rabbit	Rb1	AnaSpec	55432	1:500	WB
Rabbit	p-Rb1 (S780)	Abcam	ab47763	1:500	WB
Rabbit	Beta-actin	Millipore	EP1123Y	1:1000	WB
Rabbit	E2f1	Abcam	ab14769	1:1000	WB

IHC: Immunohistochemistry; PCNA: Proliferating cell nuclear antigen; WB: Western blot.

### Fin regeneration and wounding

Caudal fin amputations were performed with a razor blade on fish anesthetized with 0.016% tricaine in aquarium water; consistently only the distal halves of fins were amputated. Heat shocks were delivered by housing fish in a water bath set to 37°C with bidiurnal water exchanges. The water bath achieved 37°C within 15 min, maintained that temperature for 1 hr, and then passively cooled to fish room temperature (26–28°C). An automatic digital timer (Intermatic, Spring Grove, IL) was used to turn on and off the water bath. For heat shock experiments, an initial heat shock was delivered and then fins were amputated 3 hr later. Heat shocks were subsequently delivered every 6 hrs for the duration of the experiment. Quantification of fin regenerate length, area, and GFP intensity was performed in ImageJ. Fin regenerate length was calculated by averaging the length of the longest dorsal and ventral fin ray from the amputation plane. Caudal fin wounding experiments were performed as previously described (Gauron et al., 2013).

### ChIP

ChIP of zebrafish fin tissue was performed as previously described (Wehner et al., 2014) with a Bioruptor UCD-200 (Diagenode, Denville, NJ) at high power for six 5-min cycles of 30 s ON, 30 s OFF; water was changed after each cycle; 5 μg of rabbit anti-E2f1 antibody or rabbit IgG (Vector Laboratories) was used.

Promoter annotation was performed by first identifying the sequences amplified by each primer set (Table 2) using the Ensembl Genome Browser and then inputting those sequences into Tfsitescan (http://www.ifti.org/). E2f binding sites were identified and highlighted in bold (Figure 2—figure supplement 1).

**Table 2.** Chromatin immunoprecipitation primers. **DOI:**http://dx.doi.org/10.7554/eLife.07702.021

Gene	Ensembl ID	Target site	Forward primer	Reverse primer
CDKN2A	ENSG00000147889	TSS	5^′^-GCTGAGGGTGGGAAGATG-3^′^	5^′^-CCTTAACTGC AGACTGGGA-3^′^
tk1	ENSDARG00000086561	TSS	5^′^-AGTCACTGTGCCGGTTTATT-3^′^	5^′^-GTCGTCTGCTTG TTGTCTTTATTT-3^′^
tk1-	ENSDARG00000086561	2 kbp 5^′^ of TSS	5^′^-CAGGCTTACGGAGACAGCAA-3^′^	5^′^-AGTGTTTGCTG CTGGATCAC-3^′^

TSS: Transcriptional state site.

### Gene quantification

qPCR assays were performed on 100 ng of cDNA using 1 μL of each primer (10 pmol/μL) and iTaq Universal SYBR Green Supermix (Bio-Rad, Hercules, CA, United States)  in a 12 μL total reaction volume (Table 3). The PCR was performed for 40 cycles with annealing temperatures of 58–60°C and elongation times of 1 min. Total RNA was isolated using the RNeasy Mini Kit (Qiagen, Netherlands) per the manufacturer’s instructions. cDNA was prepared from total RNA using random hexamer primers and the SuperScript III First Stand Synthesis System for reverse transcription-PCR (Life Technologies) per the manufacturer’s instructions. Primers used to quantify *tp53* and *cdkn1a* expression levels have been previously described (Danilova et al., 2014).

**Table 3.** Quantitative polymerase chain reactionprimers. **DOI:**http://dx.doi.org/10.7554/eLife.07702.022

Gene	RefSeq ID	Forward primer	Reverse primer
CDKN2A	NM_058195.3	5^′^-ATGGTGCGCAGGTTCTTGGTGA-3^′^	5^′^-CACCACCAGCGTGT CCAGGAAG-3^′^
actb2	NM_181601.4	5^′^-CGAGCAGGAGATGGGAACC-3^′^	5^′^-CAACGGAAACGCT CATTGC-3^′^
tp53	NM_131327.2	5^′^-CTGAAGTGGTCCGCAGATG-3^′^	5^′^-CGTTTGGTCCCAG TGGTGG-3^′^
cdkn1a	NM_001128420.1	5^′^-AGCTGCATTCGTCTCGTAGC-3^′^	5^′^-TGAGAACTTACT GGCAGCTTCA-3^′^

### Cell culture

Zebrafish cells were cultured at 32°C, 5% CO_2_ in Dulbecco’s Modified Eagle Medium: Nutrient Mixture F-12 (DMEM:F-12) medium (ATCC, Manassas, VA) with 10% fetal bovine serum (FBS), 1% penicillin/streptomycin (Pen/Strep) (ZF4), or 50% L-15, 35% DMEM, 15% Ham’s F-12 medium with 1.8 mM NaHCO_3_, 15 mM HEPES, 1% Pen/Strep, 10% FBS, 1% l-glutamine, 0.2% gentamicinsulfate (ZKS). HeLa cells were grown at 37°C, 5% CO_2_ in DMEM with 10% FBS, 1% Pen/Strep. The pcDNA-ARF construct was created by subcloning the cDNA of human ARF (exons 1β, 2, and 3 of *CDKN2A*) into the multiple cloning site of pcDNA3.1(+). Cells were transfected with either pcDNA-ARF or an empty vector (pcDNA). Transient transfections were performed using the FuGENE 6 transfection reagent (Promega, Madison, WI) according to the manufacturer’s instructions. Cells were analyzed 2 days posttransfection. Luciferase assays were performed with pGL3-ARF-736 bp and pGL3-ARF-3.4 kb as previously described (del Arroyo et al., 2007) without activator DNA.

### Statistical analysis

Data are presented as mean ± standard deviation. Statistical analyses were performed by using SPSS Statistics Desktop, version 22.0 (IBM, Armonk, NY). Statistical differences were analyzed by using a Student’s *t*-test. A p<0.05 was set as the threshold for statistical significance.

## References

[bib1] Belyi VA, Ak P, Markert E, Wang H, Hu W, Puzio-Kuter A, Levine AJ (2010). The origins and evolution of the p53 family of genes. Cold Spring Harbor Perspectives in Biology.

[bib2] Berghmans S, Murphey RD, Wienholds E, Neuberg D, Kutok JL, Fletcher CDM, Morris JP, Liu TX, Schulte-Merker S, Kanki JP, Plasterk R, Zon LI, Look AT (2005). Tp53 mutant zebrafish develop malignant peripheral nerve sheath
tumors. Proceedings of the National Academy of Sciences of the United States of
America.

[bib3] Berman SD, Yuan TL, Miller ES, Lee EY, Caron A, Lees JA (2008). The retinoblastoma protein tumor suppressor is important for
appropriate osteoblast differentiation and bone development. Molecular Cancer Research : MCR.

[bib4] Brockes JP, Kumar A (2008). Comparative aspects of animal regeneration. Annual Review of Cell and Developmental Biology.

[bib5] Chin L, Pomerantz J, DePinho RA (1998). The INK4a/ARF tumor suppressor: one gene—two products—two
pathways. Trends in Biochemical Sciences.

[bib6] Chitramuthu BP, Bennett HPJ (2013). High resolution whole mount in situ hybridization within zebrafish
embryos to study gene expression and function. Journal of Visualized Experiments : JoVE.

[bib7] Clark LD, Clark RK, Heber-Katz E (1998). A new murine model for mammalian wound repair and
regeneration. Clinical Immunology and Immunopathology.

[bib8] Damalas A, Velimezi G, Kalaitzakis A, Liontos M, Papavassiliou AG, Gorgoulis V, Angelidis C (2011). Loss of p14(ARF) confers resistance to heat shock- and oxidative
stress-mediated cell death by upregulating β-catenin. International Journal of Cancer. Journal International Du Cancer.

[bib9] Danilova N, Bibikova E, Covey TM, Nathanson D, Dimitrova E, Konto Y, Lindgren A, Glader B, Radu CG, Sakamoto KM, Lin S (2014). The role of the DNA damage response in zebrafish and cellular models
of diamond blackfan anemia. Disease Models & Mechanisms.

[bib10] del Arroyo AG, El Messaoudi S, Clark PA, James M, Stott F, Bracken A, Helin K, Peters G (2007). E2F-dependent induction of p14ARF during cell cycle re-entry in human
t cells. Cell Cycle (Georgetown, Tex.).

[bib11] Flicek P, Amode MR, Barrell D, Beal K, Billis K, Brent S, Carvalho-Silva D, Clapham P, Coates G, Fitzgerald S, Gil L, Giron CG, Gordon L, Hourlier T, Hunt S, Johnson N, Juettemann T, Kahari AK, Keenan S, Kulesha E, Martin FJ, Maurel T, McLaren WM, Murphy DN, Nag R, Overduin B, Pignatelli M, Pritchard B, Pritchard E, Riat HS, Ruffier M, Sheppard D, Taylor K, Thormann A, Trevanion SJ, Vullo A, Wilder SP, Wilson M, Zadissa A, Aken BL, Birney E, Cunningham F, Harrow J, Herrero J, Hubbard TJP, Kinsella R, Muffato M, Parker A, Spudich G, Yates A, Zerbino DR, Searle SMJ (2014). Ensembl 2014. Nucleic Acids Research.

[bib12] Gauron C, Rampon C, Bouzaffour M, Ipendey E, Teillon J, Volovitch M, Vriz S (2013). Sustained production of ROS triggers compensatory proliferation and is
required for regeneration to proceed. Scientific Reports.

[bib13] Gemberling M, Bailey TJ, Hyde DR, Poss KD (2013). The zebrafish as a model for complex tissue
regeneration. Trends in Genetics : TIG.

[bib14] Gil J, Peters G (2006). Regulation of the INK4b-ARF-INK4a tumour suppressor locus: all for one
or one for all. Nature Reviews. Molecular Cell Biology.

[bib15] Gilley J, Fried M (2001). One INK4 gene and no ARF at the fugu equivalent of the human
INK4A/ARF/INK4B tumour suppressor locus. Oncogene.

[bib16] Greaves M (2007). Darwinian medicine: a case for cancer. Nature Reviews. Cancer.

[bib17] Gromley A, Churchman ML, Zindy F, Sherr CJ (2009). Transient expression of the arf tumor suppressor during male germ cell
and eye development in arf-cre reporter mice. Proceedings of the National Academy of Sciences of the United States of
America.

[bib18] Halloran MC, Sato-Maeda M, Warren JT, Su F, Lele Z, Krone PH, Kuwada JY, Shoji W (2000). Laser-induced gene expression in specific cells of transgenic
zebrafish. Development (Cambridge, England).

[bib19] Heber-Katz E, Zhang Y, Bedelbaeva K, Song F, Chen X, Stocum DL (2013). Cell cycle regulation and regeneration. Current Topics in Microbiology and Immunology.

[bib20] Jacks T, Fazeli A, Schmitt EM, Bronson RT, Goodell MA, Weinberg RA (1992). Effects of an rb mutation in the mouse. Nature.

[bib21] Kamijo T, Zindy F, Roussel MF, Quelle DE, Downing JR, Ashmun RA, Grosveld G, Sherr CJ (1997). Tumor suppression at the mouse INK4a locus mediated by the alternative
reading frame product p19ARF. Cell.

[bib22] Kim S-H, Mitchell M, Fujii H, Llanos S, Peters G (2003). Absence of p16INK4a and truncation of ARF tumor suppressors in
chickens. Proceedings of the National Academy of Sciences of the United States of
America.

[bib23] Knopf F, Hammond C, Chekuru A, Kurth T, Hans S, Weber CW, Mahatma G, Fisher S, Brand M, Schulte-Merker S, Weidinger G (2011). Bone regenerates via dedifferentiation of osteoblasts in the zebrafish
fin. Developmental Cell.

[bib24] Komarov PG, Komarova EA, Kondratov RV, Christov-Tselkov K, Coon JS, Chernov MV, Gudkov AV (1999). A chemical inhibitor of p53 that protects mice from the side effects
of cancer therapy. Science.

[bib25] Kwan KM, Fujimoto E, Grabher C, Mangum BD, Hardy ME, Campbell DS, Parant JM, Yost HJ, Kanki JP, Chien C-B (2007). The Tol2kit: a multisite gateway-based construction kit for Tol2
transposon transgenesis constructs. Developmental Dynamics : An Official Publication of the American Association
of Anatomists.

[bib26] Lowe SW, Sherr CJ (2003). Tumor suppression by Ink4a–arf: progress and puzzles. Current Opinion in Genetics & Development.

[bib27] MacDonald R, Guille M (1999). Zebrafish Immunohistochemistry. Molecular Methods in Developmental Biology.

[bib28] MacDonald RB, Debiais-Thibaud M, Talbot JC, Ekker M (2010). The relationship between dlx and gad1 expression indicates highly
conserved genetic pathways in the zebrafish forebrain. Developmental Dynamics.

[bib29] Menéndez S, Khan Z, Coomber DW, Lane DP, Higgins M, Koufali MM, Lain S (2003). Oligomerization of the human ARF tumor suppressor and its response to
oxidative stress. The Journal of Biological Chemistry.

[bib30] Molofsky AV, He S, Bydon M, Morrison SJ, Pardal R (2005). Bmi-1 promotes neural stem cell self-renewal and neural development
but not mouse growth and survival by repressing the p16Ink4a and p19Arf senescence
pathways. Genes & Development.

[bib31] Monaghan JR, Maden M (2013). Cellular plasticity during vertebrate appendage
regeneration. Current Topics in Microbiology and Immunology.

[bib32] Morgan TH (1901). Regeration and liability to injury. Science (New York, N.Y.).

[bib33] Muneoka K, Allan CH, Yang X, Lee J, Han M (2008). Mammalian regeneration and regenerative medicine. Birth Defects Research. Part C, Embryo Today : Reviews.

[bib34] Pajcini KV, Corbel SY, Sage J, Pomerantz JH, Blau HM (2010). Transient inactivation of rb and ARF yields regenerative cells from
postmitotic mammalian muscle. Cell Stem Cell.

[bib35] Pardal R, Molofsky AV, He S, Morrison SJ (2005). Stem cell self-renewal and cancer cell proliferation are regulated by
common networks that balance the activation of proto-oncogenes and tumor
suppressors. Cold Spring Harbor Symposia on Quantitative Biology.

[bib36] Pearson BJ, Sánchez Alvarado A (2008). Regeneration, stem cells, and the evolution of tumor
suppression. Cold Spring Harbor Symposia on Quantitative Biology.

[bib37] Pomerantz J, Schreiber-Agus N, Liégeois NJ, Silverman A, Alland L, Chin L, Potes J, Chen K, Orlow I, Lee HW, Cordon-Cardo C, Depinho RA (1998). The Ink4a tumor suppressor gene product, p19Arf, interacts with MDM2
and neutralizes MDM2's inhibition of p53. Cell.

[bib38] Pomerantz JH, Blau HM (2013). Tumor suppressors: enhancers or suppressors of
regeneration?. Development (Cambridge, England).

[bib39] Poss KD (2010). Advances in understanding tissue regenerative capacity and mechanisms
in animals. Nature Reviews. Genetics.

[bib40] Poss KD, Keating MT, Nechiporuk A (2003). Tales of regeneration in zebrafish. Developmental Dynamics : An Official Publication of the American Association
of Anatomists.

[bib41] Robertson KD, Jones PA (1998). The human ARF cell cycle regulatory gene promoter is a CpG island
which can be silenced by DNA methylation and down-regulated by wild-type
p53. Molecular and Cellular Biology.

[bib42] Rodier F, Campisi J, Bhaumik D (2007). Two faces of p53: aging and tumor suppression. Nucleic Acids Research.

[bib43] Serrano M, Lee H-W, Chin L, Cordon-Cardo C, Beach D, DePinho RA, Serrano M (1996). Role of the INK4a locus in tumor suppression and cell
mortality. Cell.

[bib44] Sharpless NE (2005). INK4a/ARF: a multifunctional tumor suppressor locus. Mutation Research.

[bib45] Sharpless NE, Depinho RA (2007). How stem cells age and why this makes us grow old. Nature Reviews. Molecular Cell Biology.

[bib46] Sherr CJ (2006). Divorcing ARF and p53: an unsettled case. Nature Reviews. Cancer.

[bib47] Smith J, Putta S, Walker J, Kump D, Samuels A, Monaghan J, Weisrock D, Staben C, Voss S (2005). Sal-site: integrating new and existing ambystomatid salamander
research and informational resources. BMC Genomics.

[bib48] Sousa S, Afonso N, Bensimon-Brito A, Fonseca M, Simoes M, Leon J, Roehl H, Cancela ML, Jacinto A (2011). Differentiated skeletal cells contribute to blastema formation during
zebrafish fin regeneration. Development.

[bib49] Stachura DL, Reyes JR, Bartunek P, Paw BH, Zon LI, Traver D (2009). Zebrafish kidney stromal cell lines support multilineage
hematopoiesis. Blood.

[bib50] Straube WL, Tanaka EM (2006). Reversibility of the differentiated state: regeneration in
amphibians. Artificial Organs.

[bib51] Tu S, Johnson SL (2011). Fate restriction in the growing and regenerating zebrafish
fin. Developmental Cell.

[bib52] Vassilev LT, Vu BT, Graves B, Carvajal D, Podlaski F, Filipovic Z, Kong N, Kammlott U, Lukacs C, Klein C, Fotouhi N, Liu EA (2004). In vivo activation of the p53 pathway by small-molecule antagonists of
MDM2. Science (New York, N.Y.).

[bib53] Weber JD, Jeffers JR, Rehg JE, Randle DH, Lozano G, Roussel MF, Sherr CJ, Zambetti GP (2000). P53-independent functions of the p19(ARF) tumor
suppressor. Genes & Development.

[bib54] Weber JD, Taylor LJ, Roussel MF, Sherr CJ, Bar-Sagi D (1999). Nucleolar arf sequesters Mdm2 and activates p53. Nature Cell Biology.

[bib55] Wehner D, Cizelsky W, Vasudevaro MD, Ozhan Günes, Haase C, Kagermeier-Schenk B, Röder A, Dorsky RI, Moro E, Argenton F, Kühl M, Weidinger G (2014). Wnt/-catenin signaling defines organizing centers that orchestrate
growth and differentiation of the regenerating zebrafish caudal
finn. Cell Reports.

[bib56] Wells J, Graveel CR, Bartley SM, Madore SJ, Farnham PJ (2002). The identification of E2F1-specific target genes. Proceedings of the National Academy of Sciences of the United States of
America.

[bib57] Wills AA, Kidd AR, Lepilina A, Poss KD (2008). Fgfs control homeostatic regeneration in adult zebrafish
fins. Development (Cambridge, England).

[bib58] Yadav VK, Kumar A, Mann A, Aggarwal S, Kumar M, Roy SD, Pore SK, Banerjee R, Mahesh Kumar J, Thakur RK, Chowdhury S (2014). Engineered reversal of drug resistance in cancer cells--metastases
suppressor factors as change agents. Nucleic Acids Research.

[bib59] Yun MH, Gates PB, Brockes JP (2013). Regulation of p53 is critical for vertebrate limb
regeneration. Proceedings of the National Academy of Sciences of the United States of
America.

[bib60] Zindy F, Williams RT, Baudino TA, Rehg JE, Skapek SX, Cleveland JL, Roussel MF, Sherr CJ (2003). Arf tumor suppressor promoter monitors latent oncogenic signals in
vivo. Proceedings of the National Academy of Sciences of the United States of
America.

