# Supplementary material for: The human ARF tumor suppressor senses blastema activity and suppresses epimorphic tissue regeneration
Source: eLife. 2015 Nov 17;4:e07702. doi: 10.7554/eLife.07702 (PMC4657621; doi:10.7554/eLife.07702)
Supplement: Related texts

# Article commentary: Decision letter

**DOI:** 10.7554/eLife.07702.023

**Affiliations:** California Institute of Technology, United States

eLife posts the editorial decision letter and author response on a selection of the published articles (subject to the approval of the authors). An edited version of the letter sent to the authors after peer review is shown, indicating the substantive concerns or comments; minor concerns are not usually shown. Reviewers have the opportunity to discuss the decision before the letter is sent (see review process). Similarly, the author response typically shows only responses to the major concerns raised by the reviewers.

Thank you for submitting your work entitled "The human ARF tumor suppressor senses blastema activity and suppresses epimorphic tissue regeneration" for peer review at *eLife*. Your submission has been favorably evaluated by Fiona Watt (Senior Editor), a Reviewing Editor, and three reviewers.

The reviewers have discussed the reviews with one another and the Reviewing Editor has drafted this decision to help you prepare a revised submission.

The following individuals responsible for the peer review of your submission have agreed to reveal their identity: Marianne Bronner (Reviewing Editor) and Gage Crump (peer reviewer).

In this manuscript, Hesse and colleagues tested human ARF promoter activity and protein function during fin regeneration. They showed that a 735 bp ARF promoter fragment drives transgene expression during fin regeneration, but not noticeably during development. Their analysis suggests that E2F1, which might be de-repressed by hyperphosphorylated-Rb1, binds ARF promoter. Next, they examined ARF protein function during fin regeneration, using a heat-shock-inducible ARF overexpression transgenic line. ARF introduction slowed fin regeneration, an effect that is partially suppressed by p53 loss-of-function mutation or inhibition of p53 activity. Furthermore, they find that ectopic expression of ARF driven by the human ARF promoter fragment inhibits fin regeneration. Overall, they provide evidence that a tumor suppressor gene ARF that is not present in zebrafish can impact regeneration, and that the Rb-E2F-ARF-p53 axis is functionally conserved among vertebrate species.

The result that the human ARF promoter is selectively activated during tail fin regeneration, but not during development or in wounding is interesting, and will likely be a useful tool for the regeneration community. The paper is well written and the experiments provide further evidence for a hypothesis that tumor suppression counteracts regeneration in mammals. However, some of their data are not as compelling as they should be. Additional basic characterizations, a better description of methods, and improved images are needed to support their interesting hypothesis. Specific criticisms are summarized below:

1) The finding that misexpression of ARF blocks tail fin regeneration by interacting with the same set of players used in tumor suppression (E2f1, Rb, p53) is a nice result (especially showing that driving ARF from the blastema-induced ARF promoter blocks regeneration). However, one wonders whether driving high-level expression of a tumor suppressor in general would have this same effect, since the usual function of these proteins is to block cell proliferation and induce cell death (as shown here for the regenerating fin). Given that loss of ARF was previously shown to enhance dedifferentiation in mice, and that reduction of p53 (a target of ARF) is required for limb regeneration in axolotls, the ARF gain-of-function results here are not that surprising.

2) In general, the experiments are well performed and accurately interpreted (with adequate numbers and statistics). It is curious that *Arf*^-/-^ mice have not been examined for enhanced digit tip, heart, or other regeneration, though doing so is clearly beyond the scope of this article. I thought the authors dealt well with the issue of loss of Arf most likely not being sufficient to significantly increase regenerative potential in mammals, though this needs to be formally tested in the future. What could be discussed and/or examined is whether ARF is expressed during mouse development. Given the inactivity of the human ARF promoter in zebrafish development, the prediction is that ARF is not developmentally expressed and is only induced by tumors in mammals.

3) In Figure 1B, why is a confocal section of the zebrafish used to characterize the GFP expression-standard would be a wide-field microscope image? In the current figure it is not even possible to really see the supposed fluorescence in the heart. Figure 1D. The authors report *ARF*:EGFP expression in the blastema. But, Figure 1C and D images are low quality and not well-annotated. There is a non uniform background that obscures the clarity and the image is not sharp Furthermore, imaging on a widefield microscope with DIC should help. The insets are fuzzy and out of focus. The lines in Figure 1D are confusing with wavy lines between the points-presumably this is not an extrapolation between the points. Why are there no error bars or background values for the GFP signal from WT fish? Also, the authors should indicate amputation planes in these images and show higher mag images to determine whether the GFP signal overlaps with MSXB and PCNA. Also, the authors should show whole mount images of uninjured and injured fins.

4) With regard the subsection “Zebrafish E2f1 binds the human ARF promoter specifically in the context of Rb hyperphosphorylation during regeneration” and Figure 2A, Rb1 and E2f1 expression patterns have not been reported during fin regeneration in zebrafish. The authors performed Western blot to identify Rb1 and E2f1 expression, but this assay cannot define where these proteins are expressed during fin regeneration. Rb1 and E2f1 should be assessed by in situ hybridization or immunohistochemistry to test if Rb1, E2f1, and *ARF:*EGFP are expressed in the same cell types during fin regeneration. How many replicates, and how do we assess significance?

5) In Figures 4–6, how do the authors measure the lengths of regenerating fin portions? It is not well described in the Methods. The zebrafish is in total about 3-4 cm long, so I doubt the accuracy of the authors' graphs indicating that the regenerating portions are 1 cm long.

6) A heat-shock every 6 hours in the authors' *hs*:ARF line seems intense (Figure 4), but the effects on regeneration appear very minor. The authors should assess longitudinal sections of the regenerating tissue by ARF in situ hybridization and/or immunofluorescence to confirm that the HS is inducing ARF in the regenerating tissue.

7) The authors report (in Figures 5 and 6) that p53 inhibition or loss-of-function of p53 can suppress impaired regeneration caused by ARF overexpression. To demonstrate that ARF overexpression stabilizes p53 level, they should examine whether p53 protein level is increased by western blot.

8) The authors describe (Figure 6A) that p53 mutations suppress an impaired fin regeneration phenotype caused by ARF overexpression. Better controls are *hs*:ARF and *tp53* mutant. They should examine *hs:*ARF, *tp53*, and *hs*:ARF, *tp53* together.

9) Expression of ARF in *ARF:*ARF during fin regeneration (Figure 6B). It does not appear that ARF localizes to the nucleus in these tissue sections. There are no pink, DAPI-positive nuclei. The authors should look at longitudinal sections at a couple of timepoints and need to indicate amputation planes.

10) In paragraph two of the subsection “ARF does not affect development but suppresses fin regeneration in response to regeneration signals” and Figure 6C, the authors mention that *ARF:*ARF fins "never regenerated completely". They should follow the regeneration at later stages (e.g. 14 dpa and 30 dpa) to see if regeneration is restored. If not, they should examine *ARF:*ARF expression after 6 dpa – it is stated earlier that *ARF:*GFP expression was turned off after 6 dpa.

11) With somewhat minor phenotypes and particularly the use of the short human promoter fragment, there is some concern about how compelling and consistent the effects are. It would be optimal to show consistent effects in a second stable line, particularly for *ARF:*ARF.

12) In Figure 6C, does p53 mutation or inhibition of p53 activity (PFTa) suppress the impaired regeneration phenotypes of *ARF:*ARF? It would be nice to examine p53 protein levels and transcript levels of p53 target genes in the *ARF:*ARF strain during fin regeneration.

13) It would be of interest for the authors to comment on why the ARF under its own promoter has a stronger negative effect on fin regeneration than the heatshock inducible version.

[Editors' note: further revisions were requested prior to acceptance, as described below.]

Thank you for resubmitting your work entitled "The human ARF tumor suppressor senses blastema activity and suppresses epimorphic tissue regeneration" for further consideration at *eLife*. Your revised article has been favorably evaluated by Fiona Watt (Senior Editor), a Reviewing Editor, and three reviewers. The manuscript has been improved but there are some remaining issues that need to be addressed before acceptance, as outlined below. In particular, we ask you to improve the quality of the figures.

1) It is difficult to tell from the images in Figure 6A what is being assessed. The standard is a longitudinal section with an indication of the amputation plane. Here, it looks like there are large regions without DAPI staining in these images and compartments of the tissues are not discernable or labeled. They do not appear to be assigning proliferation events to epidermis or blastema, but rather the 'regenerate'. Based on images like these, how can one be confident about quantification of proliferation or the defect they report? The authors should provide better quality data if they are to make conclusions. Same with Figure 7–figure supplement 3.

2) Histology quality in Figure 7B is poor. There appear to be areas of DAPI signals missing and it is hard to discern the structures. Showing the bright-field images of the fins for all sections might help, although normally it is not necessary. The authors need to provide publication-quality data here.

3) In Figure 7–figure supplement 3A, the authors show a few nuclei at high magnification but it is unclear what is being assessed – are these in the wound epidermis or blastema? Why is it necessary to focus on just a few cells, and where are these located in the fins with respect to the amputation plane?

# Reply: Author response

**DOI:** 10.7554/eLife.07702.024

 1) The finding that misexpression of ARF blocks tail fin regeneration by interacting with the same set of players used in tumor suppression (E2f1, Rb, p53) is a nice result (especially showing that driving ARF from the blastema-induced ARF promoter blocks regeneration). However, one wonders whether driving high-level expression of a tumor suppressor in general would have this same effect, since the usual function of these proteins is to block cell proliferation and induce cell death (as shown here for the regenerating fin). Given that loss of ARF was previously shown to enhance dedifferentiation in mice, and that reduction of p53 (a target of ARF) is required for limb regeneration in axolotls, the ARF gain-of-function results here are not that surprising. 

We agree that the most surprising finding of this work relates to the selective activity of the ARF promoter during regeneration and not during development or wound healing, rather than to the growth inhibitory effects of the ARF protein per se. Since ARF does not normally exist in fish, it was unclear whether the Rb/p53 pathway is mechanistically conserved to the degree required to recapitulate ARF functions when introduced into that species. Therefore, in contrast to most other tumor suppressors, which have conserved orthologues in fish and other vertebrates, the possibility of studying the impact of ARF on regenerative capacity over evolutionary distances was unclear and turned out to be feasible. We agree that driving other tumor suppressors using strong inducible or constitutive promoters during regeneration would in general be expected to inhibit the proliferation dependent aspects of the process. However, some tumor suppressors such as Rb or Hippo, which are essential for tissue formation and differentiation are probably required for effective regeneration, and modulation could conceivably enhance regeneration, a line of experimentation which merits investigation in our opinion. We address these comments in the Discussion section.

* 2) In general, the experiments are well performed and accurately interpreted (with adequate numbers and statistics). It is curious that *Arf^-/-^* mice have not been examined for enhanced digit tip, heart, or other regeneration, though doing so is clearly beyond the scope of this article. I thought the authors dealt well with the issue of loss of Arf most likely not being sufficient to significantly increase regenerative potential in mammals, though this needs to be formally tested in the future. What could be discussed and/or examined is whether ARF is expressed during mouse development. Given the inactivity of the human ARF promoter in zebrafish development, the prediction is that ARF is not developmentally expressed and is only induced by tumors in mammals.*

We thank the reviewers for the comment. The finding of this study, that ARF is not significantly expressed during development in transgenic fish, is in good agreement with previous data in mice that that ARF is not developmentally expressed in the vast majority of mouse tissues. In the revised manuscript, we have added this to the Discussion section, citing prior work done by the Sherr laboratory using ARF reporter mice. Our similar findings in zebrafish support the fidelity of the ARF promoter used in our study. We agree with the reviewers that examination of regeneration in *Arf^-/-^*mice will be an important complement to the present study and this investigation is underway.

* 3) In Figure 1B, why is a confocal section of the zebrafish used to characterize the GFP expression-standard would be a wide-field microscope image? In the current figure it is not even possible to really see the supposed fluorescence in the heart. Figure 1D. The authors report *ARF:*EGFP expression in the blastema. But, Figure 1C and D images are low quality and not well-annotated. There is a non uniform background that obscures the clarity and the image is not sharp Furthermore, imaging on a widefield microscope with DIC should help. The insets are fuzzy and out of focus. The lines in Figure 1D are confusing with wavy lines between the points-presumably this is not an extrapolation between the points. Why are there no error bars or background values for the GFP signal from WT fish? Also, the authors should indicate amputation planes in these images and show higher mag images to determine whether the GFP signal overlaps with MSXB and PCNA. Also, the authors should show whole mount images of uninjured and injured fins.*

We acknowledge the reviewers’ comments, and the revised figure is significantly improved. In the revised manuscript we have replaced Figure 1B with the standard wide-field microscope images and moved the confocal images to Figure 1–figure supplement 2. Confocal images for Figure 1B were used because the confocal images have lower background in this assay which required longer exposures to demonstrate that GFP is not expressed throughout the embryo. The wide-field microscope images show the same staining pattern as the confocal images. The GFP fluorescence in the heart is readily visible at developmental time points 48 and 72 hpf. At 24 hpf, developmental expression of *cmlc2* is much lower, making the marker difficult to see at that stage. In addition to GFP immunofluorescence, we separately assayed GFP expression by in situ hybridization to attain sufficient sensitivity to support the conclusion that ARF expression is minimal if present at all during development. The images included in Figure 1C have been improved to remove uneven background and increase contrast. In Figure 1D, the requested figure modifications including annotations and amputation planes are now provided, and higher magnification images are shown in Figure 1–figure supplement 2B. The insets included in Figure 1–figure supplement 2B display the overlap of GFP, Msxb, and PCNA. Figure 1E has been revised, and the data are now depicted with the standard line chart parameters. With respect to Figure 1E, in light of the reviewers’ comments, we have clarified that there is no background level for the wild-type fish because GFP intensity of *ARF:*GFP fish was recorded relative to that of WT. The red bar has been removed because it was confusing, and we describe the measurement procedure in the legend. Additionally, we have included whole mount images of endogenous GFP expression over the whole time course from 0 hpa to 144 hpa in Figure 1–figure supplement 2C.

* 4) With regard the subsection “Zebrafish E2f1 binds the human ARF promoter specifically in the context of Rb hyperphosphorylation during regeneration” and Figure 2A, Rb1 and E2f1 expression patterns have not been reported during fin regeneration in zebrafish. The authors performed Western blot to identify Rb1 and E2f1 expression, but this assay cannot define where these proteins are expressed during fin regeneration. Rb1 and E2f1 should be assessed by in situ hybridization or immunohistochemistry to test if Rb1, E2f1, and *ARF:*EGFP are expressed in the same cell types during fin regeneration. How many replicates, and how do we assess significance?*

Since regulation of Rb1 and E2f1 is primarily by phosphorylation of Rb1 (hyperphosphorylated Rb1 (p-Rb1) is only expressed in cells entering S-phase) which in turn controls E2f1 activity, we addressed this comment by performing immunostaining of uninjured and 2 dpa *ARF:*GFP fins to visualize p-Rb1, Msxb, and GFP protein expression. The results show that very little p-Rb1 is present in the uninjured fin, in contrast to high levels of p-Rb1 in Msxb + / GFP + cells in the blastema at 2 dpa. This staining pattern is exclusive in the blastema and not the surrounding epithelium. We have included these images in the revised Figure 2, and the data is discussed in the revised text in subsection “Zebrafish E2f1 binds the human ARF promoter specifically in the context of Rb hyperphosphorylation during regeneration,” confirming that the alterations of Rb1 protein phosphorylation shown by Western occur in the blastema. In Figure 2A, a representative Western blot of 3 similar biologically independent experiments is shown. The revised graph shows quantification of 3 independent biological replicate experiments with statistics now described in the figure and the legend.

 5) In Figures 4–6, how do the authors measure the lengths of regenerating fin portions? It is not well described in the Methods. The zebrafish is in total about 3-4 cm long, so I doubt the accuracy of the authors' graphs indicating that the regenerating portions are 1 cm long.

We thank the reviewers for identifying this inaccuracy. We corrected the scale and the revised images and graphs to reflect the correction. We have also included a more detailed description of how regenerating fin lengths were measured in the subsection “Fin Regeneration & Wounding”.

* 6) A heat-shock every 6 hours in the authors' *hs*:ARF line seems intense (Figure 4), but the effects on regeneration appear very minor. The authors should assess longitudinal sections of the regenerating tissue by ARF in situ hybridization and/or immunofluorescence to confirm that the HS is inducing ARF in the regenerating tissue.*

In the revised manuscript, we now show immunostaining for ARF on longitudinal sections of 4 dpa WT and *hs*:ARF fins. The data are shown in Figure 4-figure supplement 1A. As expected ARF expression is detected in regenerating tissue of *hs*:ARF fin but not WT fins. Although the heat shock every 6 hours is intense from the heat shock standpoint, the short half-life of the ARF protein results in fluctuating ARF expression, rather than sustained high levels. Nonetheless, the inhibitory effects on regeneration were consistent and readily detectable. The pulsatile expression, in contrast to that of the ARF-ARF line most likely accounts for the less severe inhibition of regeneration in the hs-ARF fish compared to ARF-ARF fish.

 7) The authors report (in Figures 5 and 6) that p53 inhibition or loss-of-function of p53 can suppress impaired regeneration caused by ARF overexpression. To demonstrate that ARF overexpression stabilizes p53 level, they should examine whether p53 protein level is increased by western blot.

In the revised manuscript, we have added data demonstrating that ARF overexpression stabilizes fish p53 levels in vivo and induces p53 target gene expression (*ckdn1a*) in the subsection “ARF suppresses fin regeneration in a p53-dependent manner by inducing apoptosis and causing cell-cycle arrest” and in Figure 4–figure supplement 1C. We show this using immunostaining and qRT-PCR rather than Western blot given the very low overall level of p53 compared to total protein (wild-type cells with ARF-induced p53 either exit the cell cycle or undergo apoptosis). We confirmed p53 protein stabilization by immunostaining 4 dpa WT and *hs*:ARF fins for p53 and ARF. We also confirmed that *tp53* and *cdkn1a* (p21) transcripts increase with ARF expression at 4 dpa relative to 0 dpa. These results are shown in the revised Figure 4–figure supplement 1.

* 8) The authors describe (Figure 6A) that p53 mutations suppress an impaired fin regeneration phenotype caused by ARF overexpression. Better controls are *hs*:ARF and *tp53* mutant. They should examine *hs*:ARF, *tp53*, and *hs*:ARF, *tp53* together.*

In the revised Figure 5A, we include images and data for the three lines examined together. The data confirm that p53 mutation rescues the ARF regeneration inhibition phenotype.

* 9) Expression of ARF in* ARF*:ARF during fin regeneration (Figure 6B). It does not appear that ARF localizes to the nucleus in these tissue sections. There are no pink, DAPI-positive nuclei. The authors should look at longitudinal sections at a couple of timepoints and need to indicate amputation planes.*

The revised Figure 7 is significantly improved with new images of uninjured and 2 dpa fin using longitudinal sections in panel B. The images clearly show nuclear ARF expression during regeneration and absence of expression in the uninjured fin. Amputation planes are indicated.

* 10) In paragraph two of the subsection “ARF does not affect development but suppresses fin regeneration in response to regeneration signals” and Figure 6C, the authors mention that *ARF*:ARF fins "never regenerated completely". They should follow the regeneration at later stages (e.g. 14 dpa and 30 dpa) to see if regeneration is restored. If not, they should examine *ARF*:ARF expression after 6 dpa – it is stated earlier that *ARF*:GFP expression was turned off after 6 dpa. *

In the revised manuscript we include Figure 7–figure supplement 2, which contains data for fin regeneration at 15 dpa and 30 dpa. The data confirm sustained regeneration inhibition. Figure 7–figure supplement 3A contains an analysis of ARF expression by immunostaining at 6 dpa in *ARF:*ARF and WT fins, showing persistent ARF expression indicating ongoing active response to and inhibition of regeneration..

* 11) With somewhat minor phenotypes and particularly the use of the short human promoter fragment, there is some concern about how compelling and consistent the effects are. It would be optimal to show consistent effects in a second stable line, particularly for *ARF:*ARF. *

We thank the reviewers for this comment. The revised manuscript clarifies that we have analyzed and found consistent phenotypes with different transgenic insertions. The revised manuscript now contains analysis of a second independent stable line that replicates the original *ARF*:ARF results. The images and regenerate length and area data have been added to Figure 7C.

* 12) In Figure 6C, does p53 mutation or inhibition of p53 activity (PFTa) suppress the impaired regeneration phenotypes of *ARF*:ARF? It would be nice to examine p53 protein levels and transcript levels of p53 target genes in the *ARF*:ARF strain during fin regeneration.*

We have addressed this comment with new experiments and the data is shown in the revised manuscript. a) We performed the PFTa experiment on WT and *ARF*:ARF fins and found that exposure to PFTa rescued fin regeneration inhibition as it did in the the *hs*:ARF line, confirming the p53 dependence of ARF inhibition of fin regeneration. This data is included in the revised Figure 7–figure supplement 3C. b) We demonstrate increased p53 protein expression by immunostaining 4 dpa WT and *ARF*:ARF fins for p53 and ARF. We also confirmed that *tp53* and *cdkn1a* (p21) transcripts significantly increase along with ARF expression at 4 dpa relative to 0 dpa. This data is shown in Figure 7–figure supplement 3B. c) We performed a proliferation analysis at 2, 4, and 6 dpa in WT and *ARF*:ARF fins using EdU staining. We used whole-mount stained fins optically sectioned using confocal z-stacks. We acknowledge the reviewers’ criticism that the previous images made it difficult to understand how Edu-positive nuclei were being quantified and the new experiment shows images of fin ray blastemas that better show separation between nuclei and confirm that the images can be quantified. The new data is shown in Figure 7–figure supplement 3D. We also used this methodology to revise the experiments done with *hs*:ARF transgenic zebrafish, which is shown in the revised Figure 6A.

 13) It would be of interest for the authors to comment on why the ARF under its own promoter has a stronger negative effect on fin regeneration than the heatshock inducible version.

*ARF*:ARF fish have a more profound phenotype because the short half life of the Arf protein results in varying ARF protein level with intermittent heat shock, rather than sustained high levels. The fluctuating expression, in contrast to that of the ARF-ARF line most likely accounts for the different severity of inhibition of regeneration in the hs-ARF fish compared to ARF-ARF fish. Presumably, the ARF promoter continuously surveils, detects and induces cell cycle exit of or eliminates aspiring blastema cells resulting in a stronger phenotype. We include these comments in the revised Discussion.

[Editors' note: further revisions were requested prior to acceptance, as described below.]

1) It is difficult to tell from the images in Figure 6A what is being assessed. The standard is a longitudinal section with an indication of the amputation plane. Here, it looks like there are large regions without DAPI staining in these images and compartments of the tissues are not discernable or labeled. They do not appear to be assigning proliferation events to epidermis or blastema, but rather the 'regenerate'. Based on images like these, how can one be confident about quantification of proliferation or the defect they report? The authors should provide better quality data if they are to make conclusions. Same with Figure 7–figure supplement 3.

The revised Figure 6A and Figure 7–figure supplement 3 include the requested presentation of representative longitudinal sections and indication of the amputation plane. The new images clearly show location of Edu incorporation. The new images should resolve any concern regarding reagent penetration and support the quantification. We also now include longitudinal sections of TUNEL analysis.

 2) Histology quality in Figure 7B is poor. There appear to be areas of DAPI signals missing and it is hard to discern the structures. Showing the bright-field images of the fins for all sections might help, although normally it is not necessary. The authors need to provide publication-quality data here.

The images in Figure 7B have been replaced. The new images are of high quality with readily discernible structures and uniform DAPI staining. The images clearly show nuclear ARF staining within and limited to the blastema of the regenerating fin.

 3) In Figure 7–figure supplement 3A, the authors show a few nuclei at high magnification but it is unclear what is being assessed – are these in the wound epidermis or blastema? Why is it necessary to focus on just a few cells, and where are these located in the fins with respect to the amputation plane? 

We thank the reviewers for pointing out that the indication of where in the fin the images were taken from was unclear. The figure (as well as Figure 5A) is now revised to precisely indicate the location, which is within the blastema. These images were added after the initial submission in response to a reviewer’s query of whether ARF is still expressed 6 days after amputation. The images clearly answer that point and show that it is, in contrast to the control. High power images were shown because they nicely show ARF expression pattern within the nucleus.
